# Liver X receptor-agonist treatment rescues degeneration in a *Drosophila* model of hereditary spastic paraplegia

**DOI:** 10.1186/s40478-022-01343-6

**Published:** 2022-03-28

**Authors:** Dwayne J. Byrne, M. Elena Garcia-Pardo, Nelson B. Cole, Belguun Batnasan, Sophia Heneghan, Anood Sohail, Craig Blackstone, Niamh C. O’Sullivan

**Affiliations:** 1 UCD School of Biomolecular and Biomedical Sciences, UCD Conway Institute, University College Dublin, Dublin 4, Ireland; 2 Cell Biology Section, Neurogenetics Branch, National Institute of Neurological Disorders and Stroke, National Institutes of Health, Bethesda, MD 20892 USA; 3 MassGeneral Institute for Neurodegenerative Disease, Massachusetts General Hospital, Charlestown, MA 02129 USA; 4 Department of Neurology, Massachusetts General Hospital and Harvard Medical School, Boston, MA 02114 USA

**Keywords:** Neurodegeneration, Endoplasmic reticulum, Lipid droplet, *Drosophila* model

## Abstract

Hereditary spastic paraplegias (HSPs) are a group of inherited, progressive neurodegenerative conditions characterised by prominent lower-limb spasticity and weakness, caused by a length-dependent degeneration of the longest corticospinal upper motor neurons. While more than 80 spastic paraplegia genes (SPGs) have been identified, many cases arise from mutations in genes encoding proteins which generate and maintain tubular endoplasmic reticulum (ER) membrane organisation. The ER-shaping proteins are essential for the health and survival of long motor neurons, however the mechanisms by which mutations in these genes cause the axonopathy observed in HSP have not been elucidated. To further develop our understanding of the ER-shaping proteins, this study outlines the generation of novel in vivo and in vitro models, using CRISPR/Cas9-mediated gene editing to knockout the ER-shaping protein ADP-ribosylation factor-like 6 interacting protein 1 (ARL6IP1), mutations in which give rise to the HSP subtype SPG61. Loss of Arl6IP1 in *Drosophila* results in progressive locomotor deficits, emulating a key aspect of HSP in patients. ARL6IP1 interacts with ER-shaping proteins and is required for regulating the organisation of ER tubules, particularly within long motor neuron axons. Unexpectedly, we identified physical and functional interactions between ARL6IP1 and the phospholipid transporter oxysterol-binding protein-related protein 8 in both human and *Drosophila* model systems, pointing to a conserved role for ARL6IP1 in lipid homeostasis. Furthermore, loss of Arl6IP1 from *Drosophila* neurons results in a cell non-autonomous accumulation of lipid droplets in axonal glia. Importantly, treatment with lipid regulating liver X receptor-agonists blocked lipid droplet accumulation, restored axonal ER organisation, and improved locomotor function in Arl6IP1 knockout *Drosophila*. Our findings indicate that disrupted lipid homeostasis contributes to neurodegeneration in HSP, identifying a potential novel therapeutic avenue for the treatment of this disorder.

**Supplementary Information:**

The online version contains supplementary material available at 10.1186/s40478-022-01343-6.

## Introduction

Hereditary spastic paraplegias (HSPs) are a group of progressive neurodegenerative conditions characterised by spasticity of the lower limb muscles due to progressive axonopathy of the longest upper motor neurons of the corticospinal tract, leading to gait impairment [40]. HSP has an estimated prevalence of 1.8–9.6/100,000, though use of different diagnostic criteria and misdiagnoses suggest that HSP prevalence is likely underestimated in many regions [38, 43, 69, 72]. Even within this range, HSP has a prevalence close to that of the more well-known motor neuron disease amyotrophic lateral sclerosis (ALS), which affects 5.4 people per 100,000 [16]. HSPs are highly clinically and genetically heterogeneous, potentially explaining some of the difficulty in accurately diagnosing the disorder. Traditionally, HSPs were described as ‘pure’ or ‘complicated’ based on the absence or presence of additional, primarily neurological, clinical symptoms [26]. With the emergence of a greater understanding of the genetics underlying HSP, categorisation increasingly relies on the causative gene mutation within the greater than 80 spastic paraplegia genes (SPG1-86), rather than the clinical presentation [9, 47]. In the absence of additional symptoms, patients typically have a normal life expectancy, but this can be affected by additional clinical features of the more complicated SPGs, leading in some patients to reduced lifespan [27]. The daunting prospect of investigating such a clinically and genetically diverse disorder is alleviated in part by molecular investigations that have revealed converging cellular functions for proteins encoded by the SPG genes. Axonal development and maintenance processes are most highly represented, with SPGs falling into one of the following groups: organelle shaping and maintenance, membrane and cargo trafficking, mitochondrial function and lipid/cholesterol metabolism [7]. Moreover, the most prevalent forms of HSP are caused by mutations in proteins which are responsible for shaping the endoplasmic reticulum (ER) membrane.

The ER is an interconnected endomembrane system extending from the nuclear envelope to the plasma membrane (PM). Across this vast cellular area, ER is morphologically and functionally compartmentalised into subdomains including helicoidal stacks of ribosome-studded ER sheets and a peripheral network of densely packed tubular structures interconnected by a vast polygonal network of tubules [52, 86, 89]. Within neurons, this tubular ER is observed as a continuous network extending the length of axons [85, 97, 99]. The ER modulates a vast array of cellular functions including lipid biogenesis and distribution, calcium storage and signalling, vesicular transport, mitochondrial and endosomal fission and biogenesis of lipid droplets (LDs), peroxisomes and autophagosomes [30, 60, 95]. The organisation of the ER is regulated by ER-shaping proteins. These proteins generally share little sequence homology; however, they all possess a hydrophobic hairpin domain by which the ER-shaping proteins insert into the ER lipid bilayer [39, 88]. By forming homomeric and heteromeric oligomers, ER-shaping proteins shape and stabilize the highly curved ER tubules [75]. Mutations in genes encoding any one of the known ER-shaping protein families causes axonopathy and gives rise to HSP, highlighting the essential role that the ER plays in the maintenance of long axons. Despite this, the specific mechanisms by which HSP-causing mutations and associated ER disruption leads to long motor neuron degeneration is not known.

ADP-ribosylation factor-like 6 interacting protein 1 (ARL6IP1) is an ER-shaping protein, mutations in which cause an autosomal-recessive form of HSP (SPG61) [54, 100]. Disease-causing variants identified include frameshift, nonsense and missense mutations, pointing to loss-of-function as the primary mechanism of pathogenicity [12, 17, 53, 54, 90]. SPG61 patients exhibit an infant-onset, complicated form of HSP accompanied by distal peripheral neuropathy, although it has recently been suggested that patients carrying homozygous nonsense mutations in *ARL6IP1* exhibit a particularly severe form of HSP [12]. While ARL6IP1 does not share primary sequence homology with the other ER-shaping proteins, it possesses two hydrophobic hairpins that are typical of ER-shaping proteins. In vitro experiments have shown that these hairpins are required for ARL6IP1 localisation to ER tubules. Overexpression of the protein drives the formation and maintenance of extensive tubulation of the ER [100] while its loss is reported to result in an increased abundance of ER sheets in the periphery of HeLa cells [24].

In this study we set out to examine the function of Arl6IP1 in novel models of SPG61 in both the fruit fly *Drosophila melanogaster* and a human cell line, to better understand how pathogenic mutations in this gene may give rise to disease. We demonstrate that ARL6IP1 has highly conserved interactions with other ER-shaping proteins and phospholipid-binding proteins. Moreover, we show that this protein functions in ER organisation and lipid homeostasis, with in vivo long motor axons particularly susceptible to loss of this gene. Finally, we show that treatment with liver X receptor (LXR)-agonists, to attenuate lipid imbalance, rescues ER disruption within axons and improves neurodegenerative phenotypes caused by loss of Arl6IP1. This work provides in vivo evidence that disruption of lipid homeostasis contributes to axonopathy caused by loss of ER-shaping proteins, offering potential as a novel therapeutic avenue for the treatment of HSP.

## Materials and methods

### *Drosophila* stocks and maintenance

Flies were maintained on standard fly food (yeast, dextrose, cornmeal and agar) under a 12 h:12 h light:dark cycle at 25 °C. Flies were transferred to fresh food 2–3 times per week. For drug treatment studies, the LXR-agonists LXR-623 (50 μg/ml, Selleckchem, Cat. No. S8390) and GW3964 (50 μg/ml, Selleckchem, Cat. No. S2630), and the antioxidant *N*-acetylcysteine amide (AD4; 40 μg/ml, Tocris, Cat. No. 5619) were dissolved in standard fly food. The same food batch with equal volume of the vehicle dimethyl sulfoxide (DMSO; Sigma-Aldrich, Cat. No. D8418) was used for the control. For knockdown experiments, the fly lines used were *w*^1118^ KK control and *UAS-Arl6IP1-RNAi* KK (line numbers 60100 and 104,790, respectively), obtained from the Vienna *Drosophila* RNAi Centre, www.vdrc.at [22] and *UAS-Orp8-RNAi* and *UAS-Vap33-RNAi* (line numbers 34743 and 27,312 respectively), obtained from the Bloomington Stock Centre [58]. Tissue-specific expression was achieved by crossing UAS lines to *da-GAL4* (Bloomington *Drosophila* Stock Center (BDSC) no. 84335); [59], *nSyb-GAL4* [10], *OK6-GAL4* [1], *repo-GAL4* (BDSC no. 7415; [74], or *nrv2-GAL4* (BDSC no. 6799; [80] as indicated in the text. Other fly stocks used were ebony double balancer stock (BDSC no. 3704), mito::GFP (BDSC no. 42737; [61], tdTomato-Sec61β (BDSC no. 64746), and Rtnl1::YFP [65].

### *Drosophila* constructs and transgenic line generation

CRISPR mutant fly lines were generated using a Co-CRISPR approach [41] with Cas9-encoding pCFD3 targeting the ebony marker gene (pCFD3-eb; Plasmid #83,380). *Arl6IP1*-targetting sgRNAs were designed using the online program available at: http://targetfinder.flycrispr.neuro.brown.edu/ [36] and were cloned into the Cas9-encoding pCFD5 vector [62] following the cloning described in [64] to produce pCFD5-Arl6IP1. Primer pairs used: *Arl6IP1*-KO-F: 5′-TGCAACAGCCATCGTGGGAGCGTA-3′; *Arl6IP1*-KO-R: 5′-AAACTACGCTCCCACGATGGCTGT-3′; *Arl6IP1*-Flag-F: 5′-TGCAGCAAAAGGAAGCTGCAGTAA-3′; *Arl6IP1*-Flag-R: 5′AAACTTACTGCAGCTTCCTTTTGC-3′. Donor DNA, consisting of the desired knockin sequence flanked by ~ 50 bp homology arms, were designed using open-access protocols as in [34, 35] (Additional file 1: Supplementary material, online resource). Ultramer DNA oligonucleotides were purchased from Integrated DNA Technologies (IDT). CRISPR mutant fly lines were generated by microinjection (performed by BestGene Inc.) of pCFD3-eb, pCFD5-Arl6IP1 and Donor DNA into yw;attP40[nos-Cas9]/CyO embryos. Injected adults were crossed to ebony double balancer stock (BDSC no. 3704) and progeny were screened for visible ebony phenotype. *Arl6IP1* CRISPR mutations were identified by Sanger sequencing (Eurofins Genomics) with knockin mutations additionally validated by western blotting.

### Quantitative PCR

Total RNA was purified from 5 to 10 third instar stage larvae in TRIzol reagent according to the manufacturer’s protocol (Invitrogen). cDNA was produced using the SuperScript III First Strand Kit (Invitrogen) and 2 µg of DNase treated RNA. Real-time quantitative PCR (qPCR) was performed using Fast SYBR® Green Real-Time PCR Master Mix (Applied Biosystems™) and analysed on a 7500 FAST real-time PCR system (Life Technologies). Rp49 was used as reference gene for each sample. The following primer pairs were used: Arl6IP1-F: 5′-CAAGTTCGAGGACGTGTGC-3′; Arl6IP1-R: 5′-AGCCAAAAGACCCAAACTCA-3′; Rp49-F: 5′-CGGATCGATATGCTAAGCTGT-3′; Rp49-R: 5′-CGACGCACTCTGTTGTCG-3′; Orp8-F: 5′-GGCCTAAAGAAGCCCTACAATC-3′; Orp8-R: 5′- CGACACCTGCTCTGCAATATAA-3′; Vap33-F: 5′- GAGAAGCAGAT TCCGGTCTTT-3′; Vap33-R: 5′-GTTGCTGGCGTTCGTTTATG-3′. Three biological replicates were conducted, and relative gene expression was calculated using the −ΔCt method, with mRNA levels from knockout or knockdown larvae normalised to those in control larvae.

### Behavioural analysis

Assessment of fly locomotion was made using a negative geotaxis assay. Age-matched male flies, separated into groups of 10 individuals per genotype, were tested together under the same conditions once a week for 5 weeks. During each experiment, the flies were transferred to empty glass vials (10 cm length, 2.5 cm diameter), knocked to the bottom of the vial by tapping against the laboratory bench three times and the proportion of flies climbing to the top of the vial over 15 s was determined. Flies were transferred to fresh fly food 1–2 times per week and mortality was measured every second day.

### Histology and immunomicroscopy

Immunostaining of *Drosophila* larvae was carried out as described previously [31]. Briefly, third-instar (L3) larvae were dissected in chilled in Calcium-free HL3 solution [79], fixed in 4% formaldehyde in phosphate-buffered saline (PBS) for 30 min, permeabilised in 0.1% Triton X-100 in PBS and incubated with the appropriate antibodies. LDs were stained with 4,4-Difluoro-1,3,5,7,8-Pentamethyl-4-Bora-3a,4a-Diaza-s-Indacene 493/503 (BODIPY™, Thermo Fisher Scientific, Cat. No. D3922). Fixed and stained preparations were mounted in Vectashield (Vector Laboratories) and viewed using an Olympus FluoView FV100 confocal microscope. Images were acquired using a 60 × /1.5 NA objective and using FV10-ASX version 04.01 software. Unless otherwise stated, axonal analyses were conducted by imaging motor axon bundles passing through segment A7 [Additional file 2: Supplementary Fig. 1, online resource]. Similarly, analyses of neuromuscular junctions (NMJs) were conducted by imaging muscles 6 and 7 at segment A7.

### Image analysis

All image analyses were carried out blind to genotype. Numbers of synaptic boutons per NMJ were counted manually, with type 1b and 1 s boutons differentiated by intensity of Discs-large (DLG) staining. To quantify tubular ER organization within axon bundles or terminal synaptic boutons at NMJs, Sec61β-tomato or Rtnl1::YFP staining was classified as continuous, i.e. having a linear staining pattern, or disrupted, i.e. staining was punctate or absent. To quantify mitochondrial area, mitochondrial circularity and LD number within axon bundles, images were first thresholded and then converted to binary images in ImageJ. The area of individual mitochondria and LDs as well as LD number (adjusted for area measured) were then determined using the “analyse particle” feature in ImageJ. Mitochondrial circularity was quantified using the Shape Descriptors option in the ImageJ/Analyze menu which uses the equation 4 π × [Area]/[Perimeter]^2^ to generate a value between 0 and 1, where 1 is a perfect circle and as the value approaches 0 it becomes increasingly elongated.

### Cell culture and transfection

Human bone osteosarcoma epithelial cells (U-2 OS) were cultured in McCoy’s 5A modified media (10% FBS, 100 µg/mL streptomycin, 100 U/mL penicillin) under standard conditions (37 °C, 5% CO_2_, 95% humidity). Avalanche-Omni Transfection Reagent (EZ Biosystems) was used for plasmid DNA transfection.

### Immunofluorescence and fluorescent microscopy

Cells were grown on coverslips to 50% confluence, fixed in 4% paraformaldehyde for 25 min at 37 °C and blocked in PBS with 4% horse serum (Sigma-Aldrich, MO, USA) and 0.1% saponin (Sigma-Aldrich) for 30 min at room temperature. For lipid droplet staining, cells were washed 3 × in PBS and incubated with 0.1 µg/mL LD540 [77] in PBS for 10 min. For immunostaining, cells were fixed as above, incubated with primary antibody in antibody dilution buffer (PBS with 2% horse serum and 0.1% saponin) overnight at 4 °C and secondary antibody for 1 h at room temperature before observation with a Zeiss LSM 880 confocal microscope with Airyscan. Images were acquired with a 63 × /1.35 NA oil immersion objective lens using the Zeiss Zen 2.3 software package.

### In vitro constructs and CRISPR gene editing

ARL6IP1-sgRNA was designed by comparing best targets identified by the online programs available at https://portals.broadinstitute.org/gppx/crispick/public [23, 70] and https://cctop.cos.uni-heidelberg.de:8043/ [78]. ARL6IP1-sgRNA was cloned into GFP-tagged Cas9-encoding vector px458 as described in [64]. Primers used:

ARL6IP1-KO-Forward: 5′-CACCGGGCTGATAAAGTCCTCCGAT-3′; ARL6IP1-KO-Reverse: 5′-AAACATCGGAGGACTTTATCAGCCC-3′; ARL6IP1-Tags-Forward: 5′-CACCGGTACATTGGAATGGCCAAGA–3′; ARL6IP1-Tags-Reverse: 5′-AAACTCTTGGCCATTCCAATGTACCC–3′. ARL6IP1 specific homology arms (600–800 bps) were generated by PCR amplification of U-2 OS genomic DNA using primers as provided in [Additional file 1: Supplementary material, online resource]. Vector segments were assembled using NEBuilder® HiFi DNA Assembly Cloning Kit (New England Biolabs) as per the manufacturer’s instructions. A sgRNA and two donor constructs [Additional file 1: Supplementary material, online resource] were used to increase the likelihood of repair, one conferring neomycin resistance and the other puromycin resistance, meaning that cells which survive dual antibiotic selection should possess at least two endogenously tagged *ARL6IP1* alleles, of which there are three in U-2 OS cells. These dsDonors were designed to tag ARL6IP1 with three small epitope tags: a mini Auxin Inducible Degron (mAID) tag used in a recently developed strategy to conditionally knockout protein expression (not pursued in this study but a useful tool for future studies [51]); a 3 × FLAG tag used for immunofluorescence and co-immunoprecipitation; and a SPOT tag, to facilitate tandem-affinity purification (TAP) in combination with the 3 × FLAG tag. U-2 OS cells were transfected with px458 encoding ARL6IP1 sgRNAs and GFP-tagged Cas9 using the XtremeGene HP DNA transfection reagent as per the manufacturer’s protocol (Sigma-Aldrich) and incubated for 72 h. Transfected U-2 OS cells were selected by fluorescence activated cell sorting (FACS) for GFP expression by the NINDS Flow Cytometry Core facility.

### Immunoblotting

Proteins were separated by SDS-PAGE on a 4–15% precast protein gel (Bio-Rad) and electrophoretically transferred to Hybond ECL membranes (Sigma-Aldrich). Membranes were blocked in 5% milk (0.1% Tween 20, Tris-buffered saline pH 7.4) for 1 h prior to incubation in primary antibody overnight at 4 °C. Following three quick washes and three 10 min washes in TBS-T (0.1% Tween 20, 150 mM NaCl, 20 mM Trisma Base), horseradish peroxidase (HRP)-conjugated secondary antibodies were added for 1 h. Finally, membranes were washed again before incubation in ECL (Thermo Fisher Scientific) and visualised on a Gel Doc XR + Gel Documentation System (Bio-Rad) and Bio-Rad Image lab software package.

### Immunoprecipitation and mass spectrometry

U-2 OS cells expressing endogenously tagged ARL6IP1 were washed three times with PBS, collected by scraping in PBS and clarified by centrifugation at 300 g for 3 min. Cells were lysed for 30 min in 1 ml of 1% NP-40 lysis buffer and centrifuged (max speed, 10 min, 4 °C). The supernatant was incubated with α-FLAG M2 affinity agarose beads (Sigma-Aldrich) for 2 h at 4 °C. Beads were washed with 1% NP-40 lysis buffer twice and 0.1% NP-40 lysis buffer twice. Bound proteins were eluted in 0.1% NP-40 lysis buffer containing 50 µg/ml 3 × FLAG Peptide for 1 h before incubation with SPOT-trap magnetic agarose beads at 4 °C for 1 h. The beads were washed four times in 0.1% NP-40 wash buffer, eluted in 2 × Laemmli buffer and ran by electrophoresis into a 7.5% Mini-PROTEAN® TGX polyacrylamide gel (Bio-Rad). Total protein was stained with 0.1% Coomassie Blue R250 solution (10% acetic acid, 50% methanol, 40% H_2_O) for 30 min and washed 4 times in destain (10% acetic acid, 50% methanol and 40% H_2_O) over 2 h. Gel lanes were cut with sterile blades and collected in microcentrifuge tubes for mass spectrometry.

Mass spectrometry (MS) analysis was conducted by Dr. Yan Li (NINDS Proteomics Core Facility, NIH, USA) based on in-gel tryptic digestion. Immunoprecipitated proteins were alkylated by 10 mM N-ethylmaleimide for 15 min and digested with trypsin for 18 h. Extracted peptides were desalted using Waters Oasis HLB 96-well plate and eluted with 100 μl of methanol. Peptides were dried in a speedvac. Samples were diluted to 5 μl by adding 0.1% trifluoroacetic acid (TFA) for MS analysis. The peptides were analysed using a Thermo Fisher Scientific Fusion Lumos mass spectrometer connected to an Ultimate Ultra3000 chromatography system incorporating an auto-sampler. The resuspended peptides were loaded onto an ES802 column (25 mm length, 75 mm ID) column and separated by an increasing acetonitrile gradient, using a 50-min reverse phase gradient at a flow rate of 300 nL/min. The mass spectrometer was operated in positive ion mode with a capillary temperature of 275 °C, with a potential of 2000 V applied to the column. Data were acquired with the mass spectrometer operating in automatic data dependent mode. Database search and label-free quantitation were performed using Proteome Discoverer.

### Antibodies

The following primary antibodies were used in this study: anti-DLG (1:50, Developmental Studies Hybridoma Bank, Cat. No. 4F3), anti-HRP (1:20, Sigma-Aldrich, Cat. No. P7899), anti-GFP (1:100 for immunohistochemistry and 1:2000 for western blot [WB], Thermo Fisher Scientific, Cat. No. A-6455), anti-actin (1:1000, Sigma, Cat. No. A4700), anti-ARL6IP1 (WB = 1:1000 and 1:100 for immunofluorescence [IF], GeneTex, Cat. No. GTX85516), anti-reticulon3 (WB = 1:1000 and IF = 1:100, Proteintech, 12055-2-AP), anti-reticulon4 (WB = 1:1000 and IF = 1:100, Proteintech, 10740-1-AP), anti-REEP5 (WB = 1:1000 and IF = 1:100, Proteintech, Cat. No. 14643-1-AP), anti-FLAG M2 (WB = 1:2000 and IF = 1:250, Sigma-Aldrich, Cat. No. F1804). Anti-rabbit (GE Healthcare, Cat. No. 95017–556) and anti-mouse (Santa Cruz Biotechnology, Cat. No. Sc-516102) horseradish peroxides-conjugated secondary antibodies were used for western blot analysis. Alexa Fluor 488/555/647 anti-rabbit (Thermo Fisher Scientific, respective Cat. No. A21206, A31572, A31573) and anti-mouse (Thermo Fisher Scientific, respective Cat. No. A21202, A31570, A31571) secondary antibodies were used for immunofluorescence.

### Statistical analysis

All data were statistically analysed using Prism 8 (GraphPad Software, Inc.). Statistical analysis of qPCR, mitochondria and LD quantification in knockout (KO) lines were conducted using one-way ANOVA and Dunnett’s post-tests. For climbing assays, two-way ANOVA and Bonferroni multiple comparisons tests were used. Survival curves were statistically analysed using the log rank Mantel-Cox test. For analysis of qPCR, ER in NMJs and LDs in knockdown lines one-way and two-way ANOVA and Bonferroni multiple comparisons tests were used as indicated. Statistical analysis of ER, mitochondria and LD quantifications in drug treated lines was conducted using two-way ANOVA and Tukey’s multiple comparisons tests. Unless otherwise specified, statistically significant differences from control are indicated throughout all graphs as *P < 0.05, **P < 0.01, ***P < 0.001, ****P < 0.0001. *P* > 0.05 is indicated as ns (not significant).

## Results

### Loss of Arl6IP1 disrupts ER and mitochondrial organisation and results in progressive locomotor dysfunction in a novel in vivo model of HSP

**Fig. 1:**
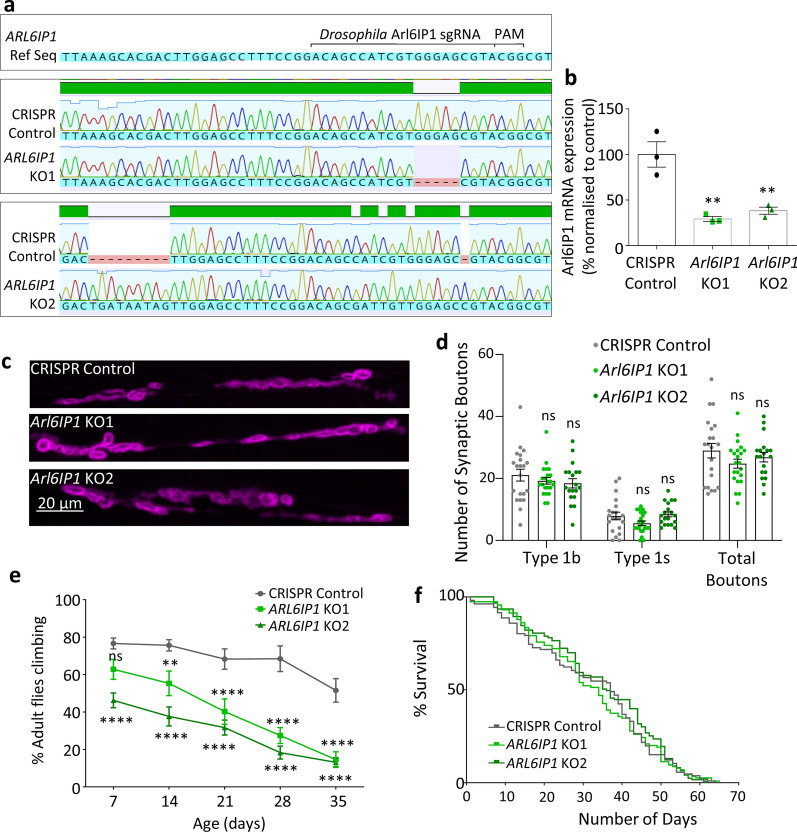
Loss of Arl6IP1 results in a progressive locomotor deficit in vivo. **a** Partial sequence of *Drosophila* Alr6IP1 showing the location of the sgRNA and PAM site used (top panel). (Lower panels) DNA sequencing results of CRISPR control and *Arl6IP1* knockout (KO) lines generated in this study aligned using Geneious software. **b** Graph represents mean ± standard error from the mean (SEM) real-time PCR analysis of Arl6IP1 expression in CRISPR control and *Arl6IP1* KO *Drosophila*. Statistical analysis consists of one-way ANOVA and subsequent Dunnett’s post-hoc tests. *n* = 3. **c** Representative confocal images of the posterior NMJ stained with the post-synaptic density marker DLG (magenta). **d** Graph represents mean ± SEM number of 1b, 1 s, and total synaptic boutons in control and *Arl6IP1* KO. *n* = 19–22 animals. Statistics consist of two-way ANOVA with Bonferroni’s post-hoc tests. **e** Arl6IP1 knockout flies exhibit locomotor deficits that progress over time. Graph represents the mean ± SEM percentage of flies that reached the top of the vial within 15 s. Statistical analysis consists of two-way ANOVA and Dunnett’s multiple comparisons tests. *n* = 13—16 independent experiments per genotype. **f** Survival assay reveals no effect of loss of Arl6IP1 on lifespan compared to controls. *n* = 115–126 flies per genotype. *p*-value = 0.6967 (ns) determined by Log-rank (Mantel-Cox) test

To investigate the cellular role of Arl6IP1, particularly within the primary neuronal structure to degenerate in HSP i.e. long motor neuron axons, we generated a novel model of SPG61 in *Drosophila melanogaster* (the common fruit fly). *Drosophila* possess a single ortholog of human *ARL6IP1* (*Arl6IP1*; CG10326) which shares 30% amino acid sequence identity [31] and is predicted to possess hydrophobic hairpin domains characteristic of the ER-shaping proteins (https://www.uniprot.org/uniprot/A0A0B4KG41). CRISPR/Cas9 mediated gene editing was employed to knock out *Drosophila Arl6IP1* using a Co-CRISPR approach [41]. Following screening, two independent lines were selected for downstream studies, a line with a 5 bp deletion (*Arl6IP1* KO1) and a knockin line (*Arl6IP1* KO2), both of which result in early stop codons, predicted to produce highly truncated proteins lacking the hairpin loop domains (Fig. 1a) [Additional file 2: Supplementary Fig. 2, online resource]. A fly line in which *Arl6IP1* had not been genetically modified by the CRISPR/Cas9 process was maintained and used as an isogenic background control line, termed CRISPR control. Unfortunately, loss of the Arl6IP1 protein could not be confirmed by western blot as neither commercially available antibodies to the human antibody, nor two *Drosophila* Arl6IP1 antibodies that we generated to two different epitopes of the protein, identified bands specific to Arl6IP1 (data not shown). However, real-time PCR revealed significantly reduced Arl6IP1 mRNA in *Arl6IP1* KO flies (Fig. 1b), suggesting that mRNA in these mutant lines is undergoing degradation due to non-sense mediated decay. *Arl6IP1* KO flies are viable, and confocal imaging of L3 stage *Drosophila* larvae revealed no gross morphological alterations to NMJs in *Arl6IP1* KO lines (Fig. 1c, d), indicating that loss of Arl6IP1 does not impact the development of neuronal synapses. As HSP is a progressive neurodegenerative condition, a negative geotaxis assay was used to examine locomotor abilities in adult flies. This revealed a significant locomotor deficit in *Arl6IP1* KO lines compared to controls, a phenotype which progressively worsened over time (Fig. 1e). Despite progressive locomotor deficits, *Arl6IP1* KO lines exhibited a survival rate comparable to that of the CRISPR control line (Fig. 1f). These phenotypes are comparable to those we have previously reported in *Arl6IP1* RNAi lines [31], though *Arl6IP1* KO flies display locomotor deficits slightly earlier than RNAi knockdown flies.

**Fig. 2:**
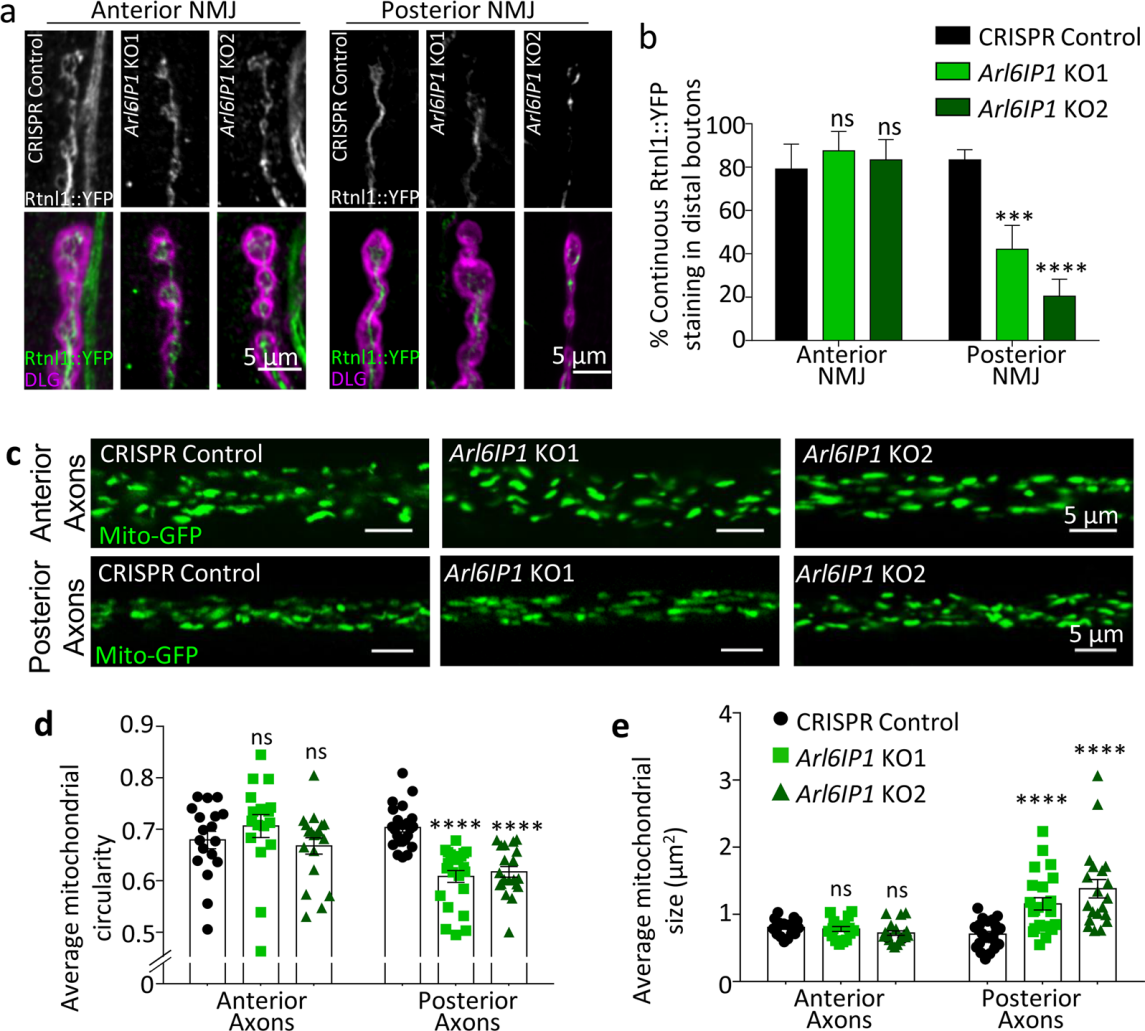
Loss of Arl6IP1 disrupts ER and mitochondrial organisation within long, but not short, motor neurons in vivo. **a** Representative confocal images of the smooth ER marker Rtnl1::YFP (green) within anterior and posterior NMJs (DLG, magenta). **b** Graph represents mean ± SEM proportion of continuous Rtnl1::YFP staining identified in CRISPR control and *Arl6IP1* knockout larvae. *n* = 19–42 animals. Statistics consist of two-way ANOVA with Tukey’s multiple comparisons test. **c** Representative confocal images of mitochondria (green) within anterior and posterior axon bundles. Graphs represent mean ± SEM mitochondrial circularity **d** and size **e** within axon bundles in *Arl6IP1* KO flies compared to controls. *n* = 18—20 animals per genotype. Statistics consist of two-way ANOVA with Tukey’s multiple comparisons test

We next examined the impact of loss of Arl6IP1 on axonal ER within motor neurons. Visualization of the endogenously expressed tubular ER-marker Reticulon-like 1 (Rtnl1)::YFP within axon terminals of the longest motor neurons revealed a marked disruption to ER organisation. In contrast to the highly continuous tubular ER staining observed in the axon terminals of control larvae, *Arl6IP1* KO lines exhibited significantly more disrupted Rtnl1::YFP distribution (Fig. 2a, b). By contrast, ER organisation is unaffected within the axons of shorter motor neurons. We have previously shown that knockdown of either Arl6IP1 or another ER-shaping protein Rtnl1 impairs mitochondrial fission resulting in disrupted axonal mitochondrial organisation [29, 31]. We therefore examined mitochondrial organisation within motor neurons of *Arl6IP1* KO *Drosophila*. Confocal microscopic analysis of mito::GFP expressed within motor neurons revealed significant elongation of mitochondria within the axons of the longest motor neurons of *Arl6IP1* KO lines (Fig. 2c–e). Notably, disruption in mitochondrial organisation was not observed in the axons of short motor neurons of *Arl6IP1* KO *Drosophila*. These findings suggest that loss of Arl6IP1 causes disruption to ER and mitochondrial organisation specifically at the distal ends of long motor neurons in vivo*.*

### Within long motor neurons, neuronal, but not glial, loss of Arl6IP1, induces cell non-autonomous LD accumulation in glia.

**Fig. 3:**
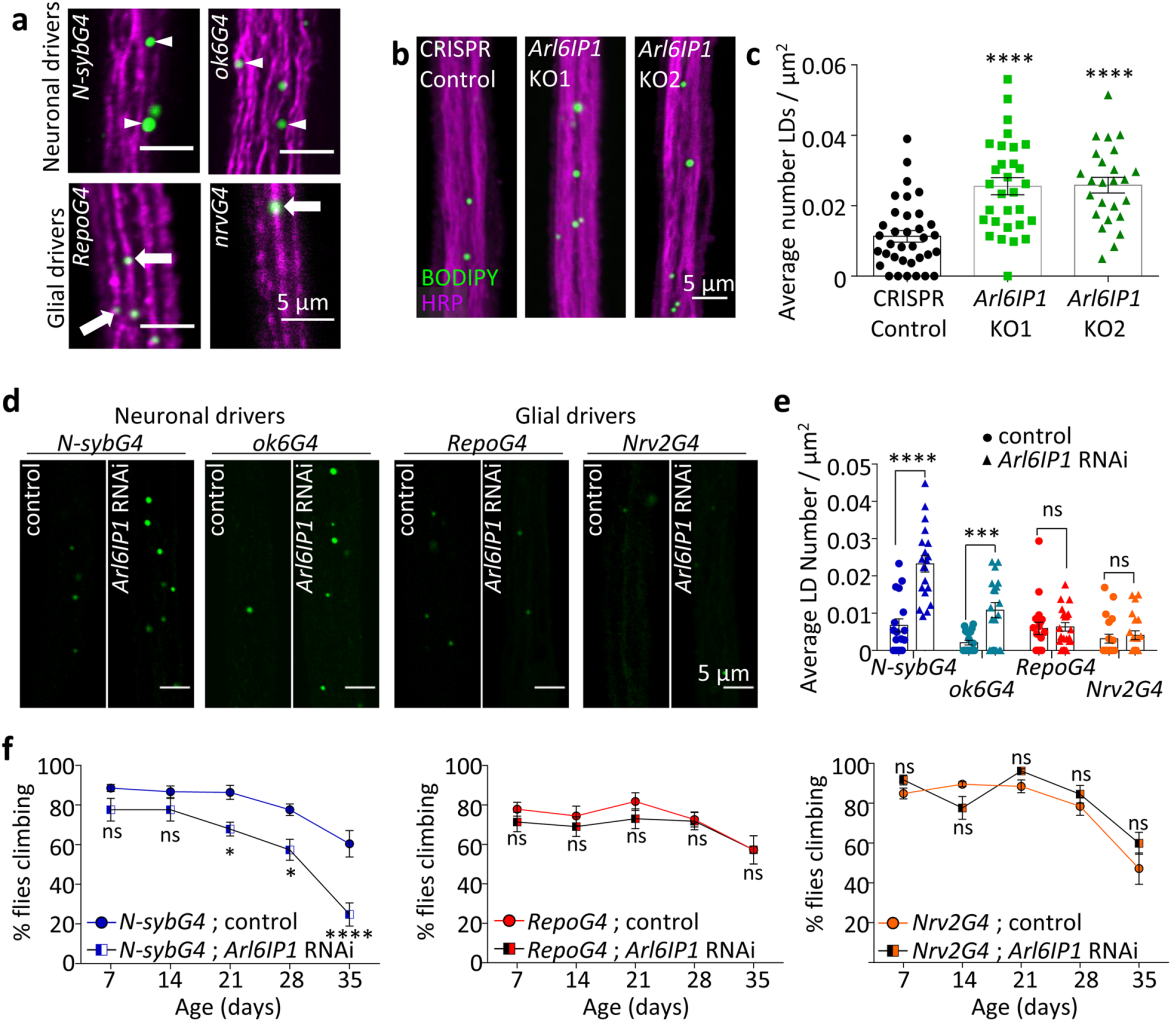
Within long motor neurons, neuronal loss of Arl6IP1 induces cell non-autonomous LD accumulation in glia. **a** Representative confocal images of BODIPY stained LDs (green) in axon bundles from flies expressing Sec61β-tomato (magenta) under the control of the indicated neuronal and glial drivers. LDs co-localise with Sec61β-tomato expressed in glia (arrows) but not neurons (arrowheads). **b** and **d** Representative confocal images of BODIPY-stained LDs (green) within posterior axon bundles **b** neuronal membranes labelled with HRP, magenta). **c** and **e** Graphs represent the mean ± SEM number of LDs per μm^2^ in the genotypes indicated. *n* = 18—35 animals per genotype. Statistics consist of **c** one-way ANOVA with Dunnett’s and **e** two-way ANOVA with Bonferroni’s multiple comparisons test. **f** Locomotor analysis of flies generated by crossing *w*^*1118*^ (control) or *Arl6IP1* RNAi to *n-SybGAL4*, *RepoGAL4* or *Nrv2GAL4* as indicated. Graphs represent mean ± SEM percentage of flies that reach the top of the vial within 15 s. *n* = 10 – 13 independent experiments per genotype. Statistics consist of two-way ANOVA with Bonferroni post-hoc test

Several lines of evidence have identified lipid homeostasis disruption in cellular and animal models of HSP (reviewed in [19, 84]). To examine whether Arl6IP1 functionally contributes to lipid homeostasis in vivo, we investigated LD organisation within axonal bundles of control and *Arl6IP1* KO flies. LDs are storage organelles, densely packed with neutral lipids, which are synthesised at and bud off from the ER (reviewed in [19, 30]. Within the nervous system, LDs predominantly localise to the glia adjacent to neurons [3]. Consistent with this, we found that BODIPY-stained LDs co-localise with glial-driven markers but not neuronal-driven markers within motor neuron axon bundles of L3 stage larvae (Fig. 3a). Further analysis revealed a significant increase in the number of LDs in the glia within axon bundles of *Arl6IP1* KO *Drosophila* compared to controls (Fig. 3b, c). Given that Arl6IP1 expression is lost in all cells in *Arl6IP1* KO *Drosophila*, we next investigated whether LD accumulation occurs as a result of loss of Arl6IP1 in neurons, glia, or both cell types, by performing tissue-specific knockdown of Arl6IP1 using the UAS-GAL4 system which induces robust reduction in Arl6IP1 expression [Additional file 2: Supplementary Fig. 3, online resource]. Immunostaining revealed that neuronal Arl6IP1 knockdown is sufficient to induce LD accumulation in axonal glia, while glial Arl6IP1 knockdown does not affect LD accumulation (Fig. 3d, e). Moreover, while knockdown of Arl6IP1 in neurons induces a progressive locomotor deficit, consistent with previous studies [29], knockdown of Arl6IP1 in glia does not affect locomotor function in *Drosophila* (Fig. 3f). Therefore, neuronal, but not glial, loss of Arl6IP1 induces cell non-autonomous LD accumulation within surrounding glia which is sufficient to cause motor neuron degeneration upon aging.

### Loss of ARL6IP1 disrupts LD organisation in a cellular model of HSP

**Fig. 4:**
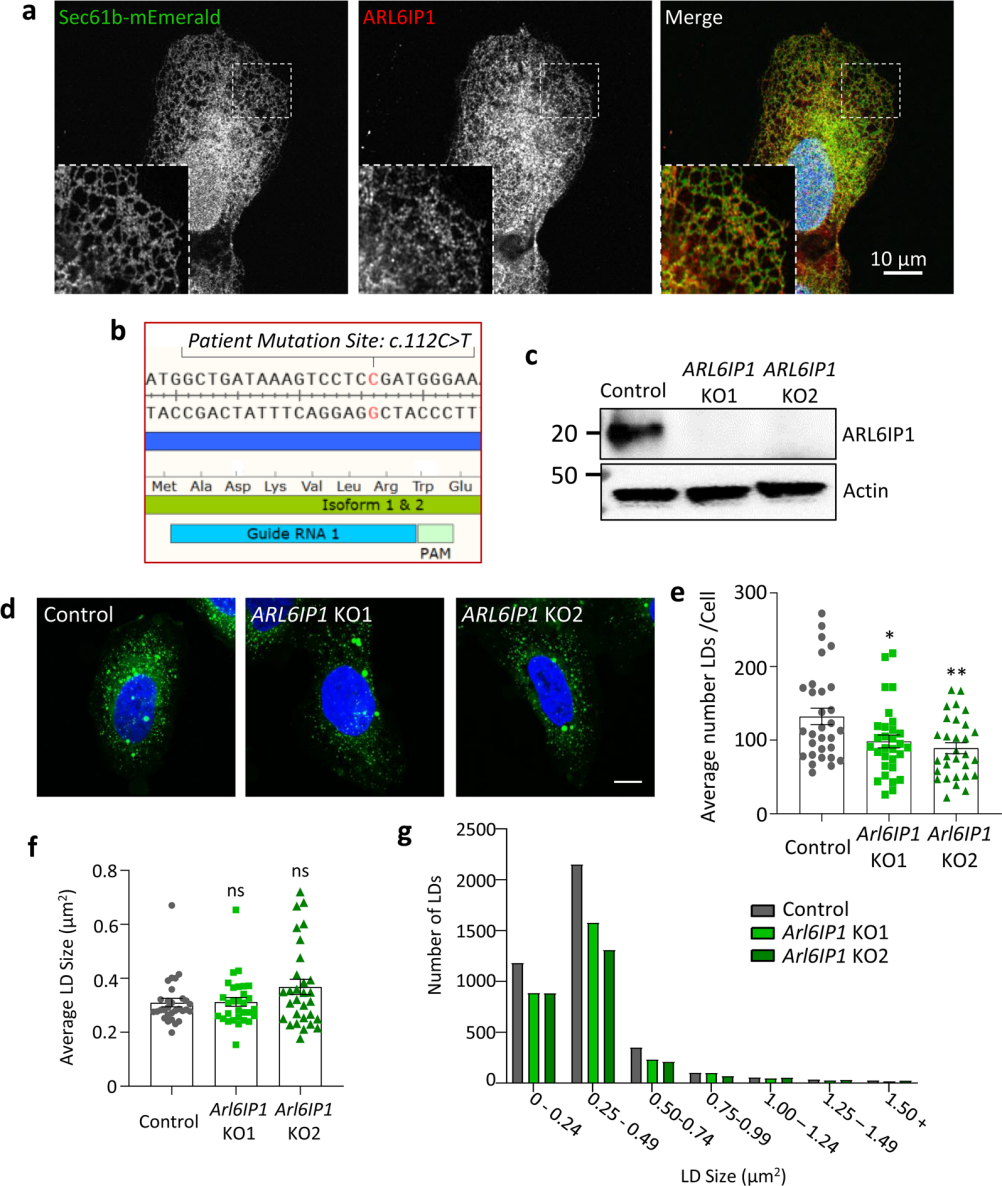
ARL6IP1 localises to the tubular ER and mediates LD organisation in vitro. **a** Representative confocal images of ARL6IP1 immunostaining (red) with ER-tubule localising protein Sec61β (green) in U-2 OS cells. ARL6IP1 localises closely with Sec61β right out to the tips of peripheral ER tubules. **b** A single guide RNA (inset) was designed against the *ARL6IP1* gene. The predicted cut site (3 bp upstream of the PAM) occurs immediately downstream of the nonsense mutation c.112C > T found to mutate an Arg residue to an early stop codon p.Arg38* in SPG61. **c** Lines generated from individual clones were screened by western blot to identify ARL6IP1 knockout lines with two independent ARL6IP1 knockout lines chosen for further experimentation. **d** Representative confocal images of LD540 stained LDs (green) in control and ARL6IP1 knockout cells. Average LD number per cell is significantly reduced by loss of ARL6IP1 (**e**) while average LD size is unaffected (**f**). Graphs represent averages from individual cells. *n* = 30 cells per genotype from 3 independent experiments. **g** Frequency distribution of LD sizes showing all LDs measured. Statistics consist of one-way ANOVA with Dunnett’s post-hoc tests

In order to identify whether ARL6IP1 has a conserved role in the regulation of lipid homeostasis beyond *Drosophila*, we set out to identify whether loss of this protein affects lipid organisation in a human cell line. In human bone osteosarcoma epithelial cells (U-2 OS), immunofluorescence staining confirmed localisation of the endogenous ARL6IP1 protein to the tubular ER, with ARL6IP1 staining observed throughout ER tubules where it exhibits strong overlap with the ER marker Sec61β in peripheral ER tubules (Fig. 4a). Given the robust and ubiquitous expression of ARL6IP1 throughout the tubular ER of U-2 OS cells, CRISPR/Cas9 mediated gene editing was undertaken to generate a novel model of HSP based on a recently identified SPG61-causing mutation (c.112C < T, p.Arg38*). This mutation induces an early stop codon which likely results in loss of the protein [90]. To generate a similar mutation, a sgRNA was designed to target the site of this mutation (Fig. 4b) in human U-2 OS cells. Single cell clones were screened by western blotting and two independent KO lines were selected for down-stream studies (*ARL6IP1* KO1 and KO2) (Fig. 4c). Next, LDs in ARL6IP1 KO and control cells were stained with LD540 and examined by immunofluorescence (Fig. 4d). A marked reduction in the number of LDs was identified in *ARL6IP1* KO cells compared to controls (Fig. 4e) while average LD size was unaffected (Fig. 4f). Examination of LD size distribution showed no gross changes to their distribution. Instead, the number of LDs decreased across most size ranges in *ARL6IP1* KOs, with a more dramatic impact on the smallest LDs (Fig. 4g). To assess whether loss of ARL6IP1 impacts large LD formation, cells were treated with oleic acid (OA). OA is a long chain, monounsaturated fatty acid that induces significant increases in cellular neutral lipids which become concentrated in LDs, through the formation of large LDs [32]. Large LD formation is unaltered at 4 or 16 h OA treatment [Additional file 2: Supplementary Fig. 4, online resource]. These findings suggest that loss of ARL6IP1 disrupts lipid homeostasis by reducing the number of constitutive LDs in U-2 OS cells.

### ARL6IP1 has a conserved interaction with the phosopholipid binding protein ORP8

**Fig. 5:**
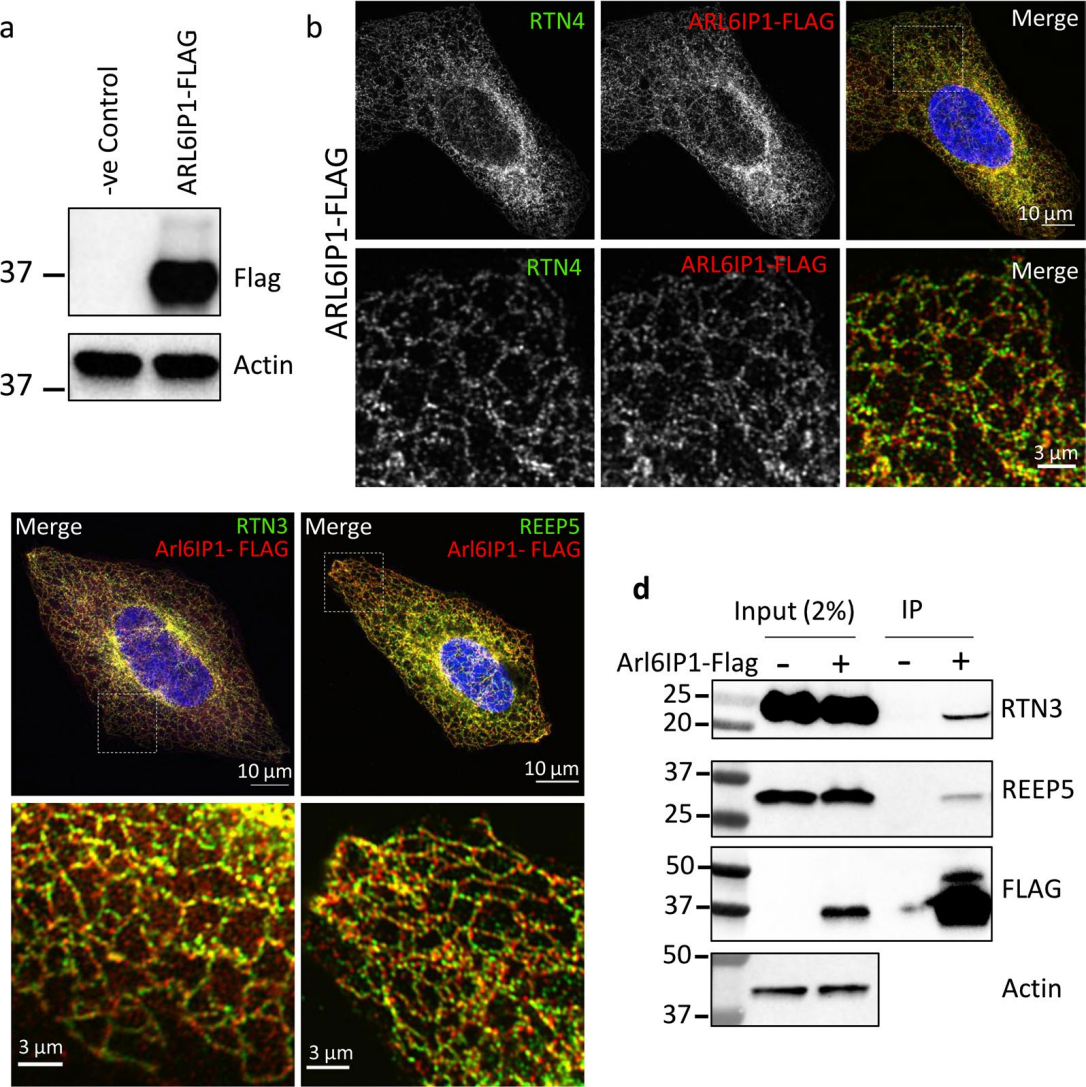
Endogenously tagged ARL6IP1 binds to ER-shaping proteins and localises to the tubular ER. **a** 3xFLAG-tagged ARL6IP1 expression was verified by western blot. **b** ARL6IP1-FLAG (red) exhibits an ER localisation pattern similar to that of untagged ARL6IP1 with strong colocalisation with the tubular ER marker, RTN4 (green). **c** Representative confocal images showing strong colocalisation between endogenously tagged ARL6IP1 and RTN3 (left) or REEP5 (right). **d** Western blot analysis of FLAG M2 affinity co-immunoprecipitated reveals interaction of ARL6IP1-FLAG with RTN3 and REEP5

To better understand its role in the regulation of lipid homeostasis, we examined the protein–protein interactors of ARL6IP1. Endogenous ARL6IP1 in U-2 OS cells was C-terminally tagged using CRISPR/Cas9 mediated gene editing [Additional file 2: Supplementary Fig. 5, online resource]. Single cell clones were screened by western blotting to identify lines in which ARL6IP1 had been successfully tagged (Fig. 5a). Immunofluorescence staining showed FLAG expression strongly colocalising with the tubular ER marker Reticulon-4 (RTN4) (Fig. 5b), consistent with the previously observed tubular ER localisation pattern observed using anti-ARL6IP1 (Fig. 4a). Next, a TAP approach consisting of a two-step co-immunoprecipitation (FLAG pull-down followed by a SPOT pull-down) of tagged ARL6IP1 and mass spectrometry analysis was conducted. U-2 OS cells in which ARL6IP1 had not been genetically modified by CRISPR/Cas9 served as negative controls to determine non-specific background protein binding. To generate a list of the likely ARL6IP1-interacting proteins, we selected only proteins that: (a) showed up in 2 of 3 independent experiments, (b) had an abundance ratio of ≥ 1.2, and c) had a combined CRAPome (a contaminant repository for AP-MS data; [48] spectral count of < 2 [98]. These thresholds and exclusion criteria generated a list of 69 proteins as likely interactors of ARL6IP1 [Additional file 3: Supplementary Table 1, online resource]. Several of these proteins had independently been identified as ARL6IP1-interactors including the ER-shaping proteins RTN4 [68] and FAM134C [46], and the phospholipid metaboliser inositol polyphosphate 5-phosphatase K (INPP5K) [24].

To identify the cellular components and functional pathways ARL6IP1 may be involved in, the list of 69 ARL6IP1 interacting proteins was entered into 4 online GO software resources: DAVID [20], Panther [49], FuncAssociate [5] and Enrichr [14]. GO analysis revealed that ARL6IP1 binding partners are highly enriched within the ER network with 29 of 69 identified as localising within the ER, ER membrane or ER tubular [Additional file 3: Supplementary Table 1, online resource]. Furthermore, investigation of the ARL6IP1-interaction network using STRING [83] revealed a large cluster of proteins linked to ER network organisation [Additional file 2: Supplementary Fig. 6, online resource]. Based on these observations, two ER-shaping proteins were chosen for further examination, namely reticulon 3 (RTN3) and receptor accessory protein 5 (REEP5). Immunostaining revealed strong colocalisation of ARL6IP1-FLAG with RTN3 and REEP5 (Fig. 5c). Both RTN3 and REEP5 also co-immunoprecipitated with ARL6IP1-FLAG (Fig. 5d), further confirming the interaction between these proteins and adding support to the experimental approach taken to identify ARL6IP1 interactors.

**Fig. 6:**
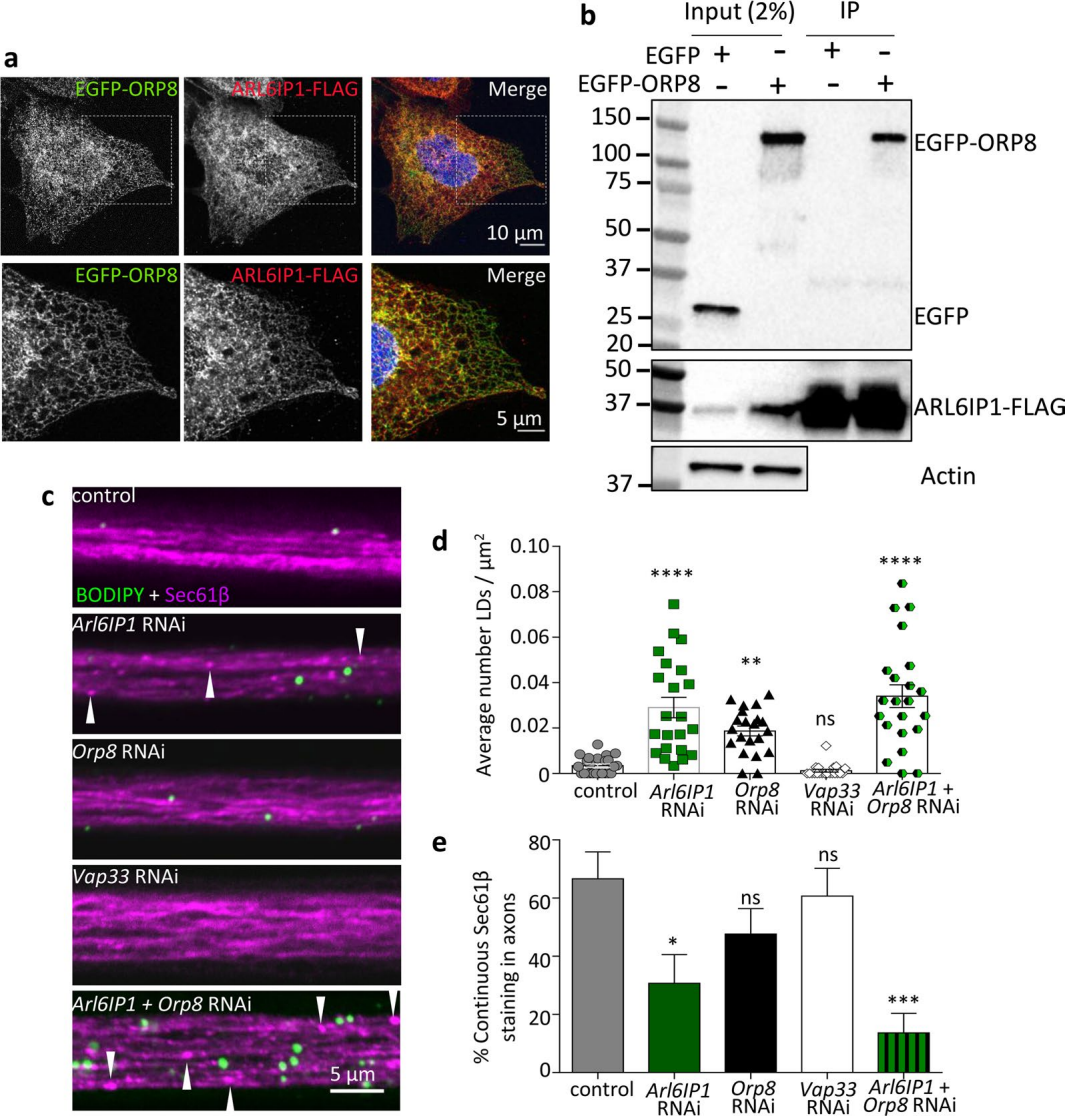
Validation of ARL6IP1 interaction with lipid transport proteins. **a** Representative confocal images showing colocalisation between endogenous FLAG-tagged ARL6IP1 and EGFP-ORP8 in U-2 OS cells. **b** Western blot analysis of FLAG M2 affinity co-immunoprecipitation showed that in ARL6IP1-FLAG expressing cells EGFP-ORP8 but not EGFP co-immunoprecipitates with ARL6IP1-FLAG. **c** Representative confocal images of BODIPY stained LDs (green) in posterior axon bundles from flies expressing Sec61β-tomato (magenta; arrowheads indicate punctate or disrupted staining) under the control of the neuronal driver *n-SybGAL4*. Graphs represent the mean ± SEM number of LDs per μm^2^ **d** and the proportion of axon bundles with continuous Sec61β-tomato staining **e** in the genotypes indicated. *n* = 21–24 animals per genotype. Statistics consist of one-way ANOVA with Bonferroni’s post-hoc tests

In addition to ER network proteins, our mass spectrometry analysis identified that ARL6IP1 interacts with several phosopholipid binding proteins including oxysterol-binding protein-related protein 8 (ORP8), which shuttles phosphoinositides between the PM and the ER [18, 33]. To verify the interaction between ARL6IP1 and ORP8, ARL6IP1-FLAG cells were transfected with EGFP-ORP8 or empty EGFP vector. Immunofluorescent examination of ARL6IP1-FLAG and EGFP-ORP8 revealed much colocalisation throughout the ER (Fig. 6a). Additionally, immunoprecipitation with FLAG M2 affinity beads revealed that EGFP-ORP8, but not EGFP alone, co-immunoprecipitates with ARL6IP1-FLAG (Fig. 6b). Taken together with the mass spectrometry data, these findings suggest that ARL6IP1 physically interacts with the lipid transport protein ORP8. We next set out to determine whether ARL6IP1-ORP8 interactions are conserved and functionally relevant in vivo. The *Drosophila* ortholog of human *ORP8* is *CG42668* (hereafter referred to as *Orp8*) which shares 55% amino acid sequence identity as revealed by a BLASTP search and contains a PH-like domain and an oxysterol-BP domain characteristic of oxysterol-binding protein family (https://www.uniprot.org/uniprot/A0A0B4JCZ0). We found that targeted knockdown of Orp8 using the UAS-GAL4 system, which induces robust reduction in Orp8 expression [Additional file 2: Supplementary Fig. 3, online resource], phenocopies the glial LD accumulation that occurs in Arl6IP1 knockdown *Drosophila* axon bundles (Fig. 6c, d). Glial LD accumulation is not observed upon targeted knockdown of all identified ARL6IP1 interactors, as evidenced by knockdown of the *Drosophila* vesicle-associated membrane protein (VAMP)-associated protein A (VAPA) ortholog *Vap33* (Fig. 6c, d). This indicates that the glial LD accumulation phenotype is specific to disruption of certain ER-phosopholipid binding proteins. Moreover, compared to control, axonal tubular ER is more disrupted in *Drosophila* in which both Arl6IP1 and Orp8 are knocked down, than in *Drosophila* in which either Arl6IP1 or Orp8 are knocked down alone (Fig. 6c, e). These observations reveal that Arl6IP1 functionally interacts with Orp8 in *Drosophila* and suggests that ARL6IP1 interactions with ER and phospholipid binding proteins are highly conserved across evolution from human to *Drosophila*.

### Treatment with LXR-agonists reduces axonal disruption and neurodegeneration in a model of SPG61

**Fig. 7:**
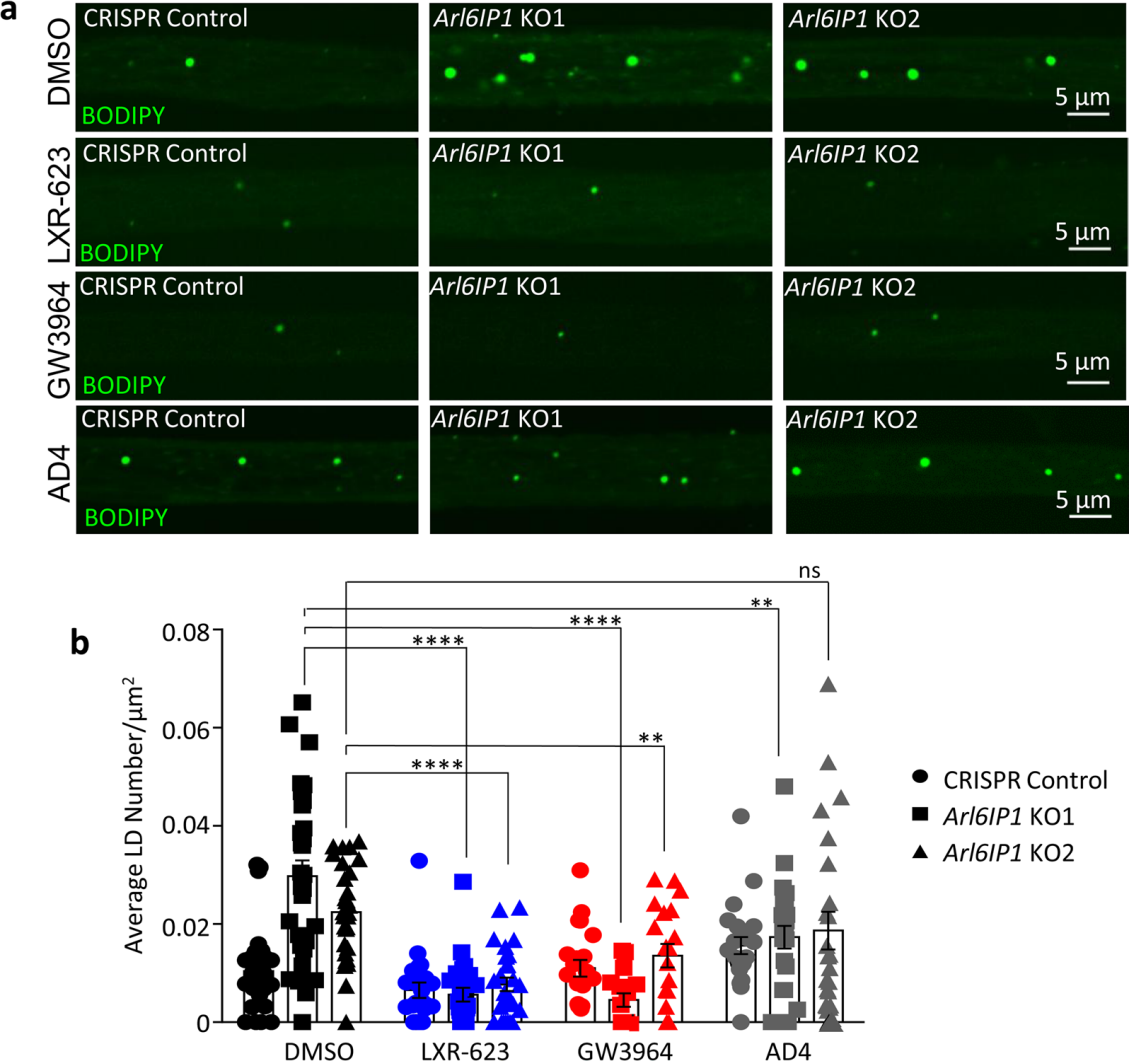
Treatment with LXR-receptor agonists rescues axonal LD accumulation caused by loss of Arl6IP1. **a** Representative confocal images of BODIPY-stained LDs (green) within posterior axon bundles from treated CRISPR Control and *Arl6IP1* knockout (KO) larvae. **b** Graphs represent the mean ± SEM number of LDs per μm^2^ in CRISPR Control and *Arl6IP1* KO larvae. *n* = 19–34 animals per genotype. Statistics consist of two-way ANOVA with Tukey’s multiple comparisons test

In order to examine whether lipid homeostasis disruption directly contributes to neurodegeneration in our *Arl6IP1* KO *Drosophila*, we used liver X-receptor (LXR) agonists to modify lipid levels. LXR-agonists activate the LXR nuclear hormone receptor superfamily of transcription factors, LXRα and LXRβ, which are expressed in the central and peripheral nervous systems where they regulate lipid and cholesterol homeostasis [13, 81, 91]. Activation of LXRs by their agonists upregulates the expression of ATP binding cassette A1 (ABCA1) and ABCG1 transporters, thereby stimulating the efflux of phospholipids and cholesterol. Of significance for this study, LXR-agonists stimulate cholesterol efflux from glia but not neurons [2, 15]. We treated *Drosophila* with two different LXR agonists (LXR-623 and GW3964) which are known to activate LXR and stimulate lipid efflux from glia [50, 87]. Chronic treatment with either drug blocked LD accumulation in *Arl6IP1* KO *Drosophila* axon bundles (Fig. 7a, b). By contrast, treatment with the antioxidant AD4, which has been shown to reduce LD accumulation within glia caused by increased reactive oxygen species (ROS) production, did not significantly affect LD accumulation in *Arl6IP1* KO axon bundles.

**Fig. 8:**
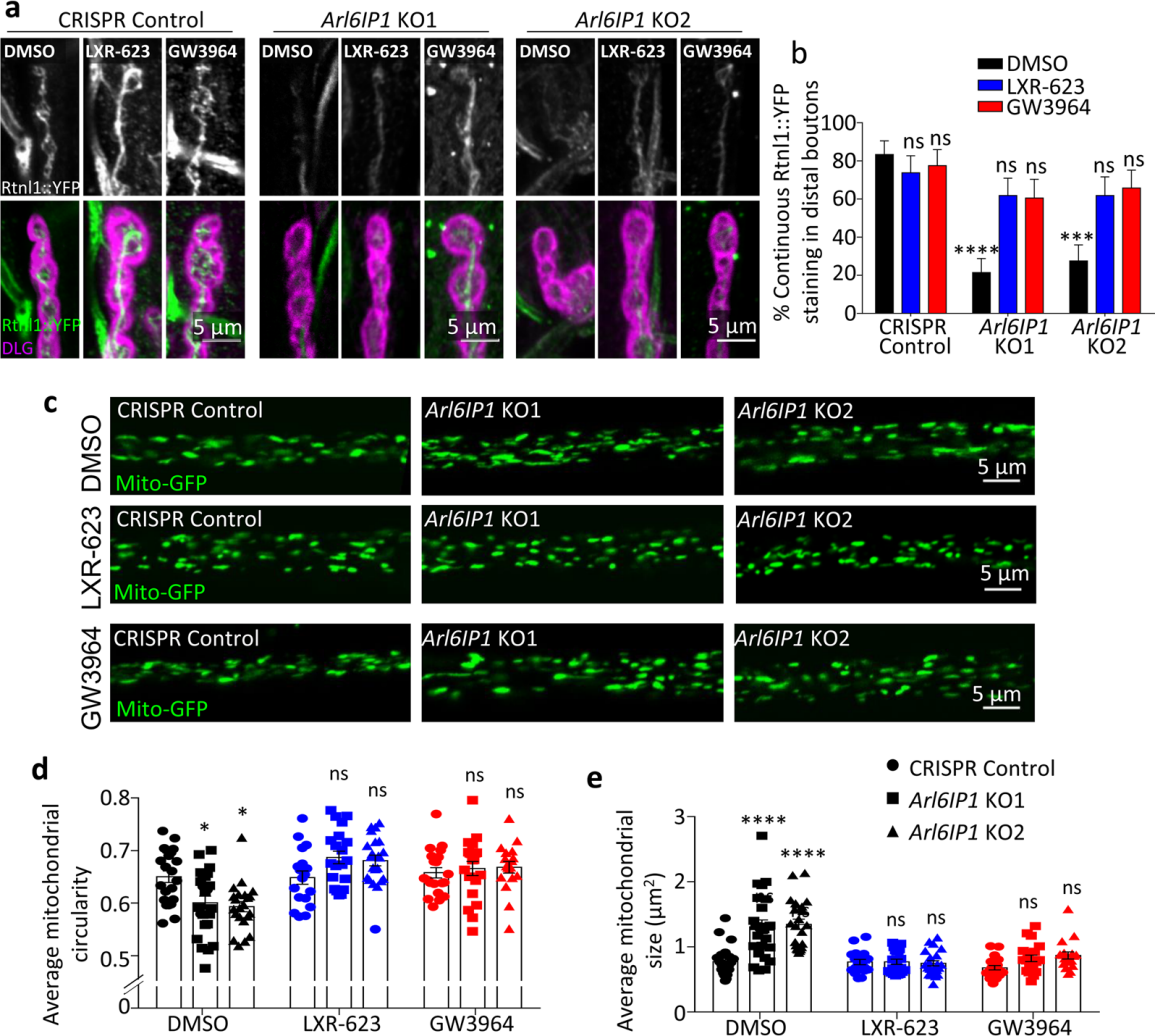
Treatment with LXR-receptor agonists improves axonal organisation in *Arl6IP1* KO models of SGP61. **a** Representative confocal images of the smooth ER marker Rtnl1::YFP (green) within posterior NMJs (DLG, magenta) from treated CRISPR Control and *Arl6IP1* KO larvae. **b** Graph represents mean ± SEM proportion of continuous Rtnl1::YFP staining identified in larval axons. *n* = 19–21 animals. Statistics consist of two-way ANOVA with Bonferroni’s multiple comparisons test. **c** Representative confocal images of mitochondria (green) within posterior axon bundles from treated CRISPR control and *Arl6IP1* KO larvae. Graphs represent mean ± SEM mitochondrial circularity **d** and size **e** within axon in CRISPR Control and *Arl6IP1* KO. *n* = 18–24 animals. Statistics consist of two-way ANOVA with Tukey’s multiple comparisons test

**Fig. 9:**
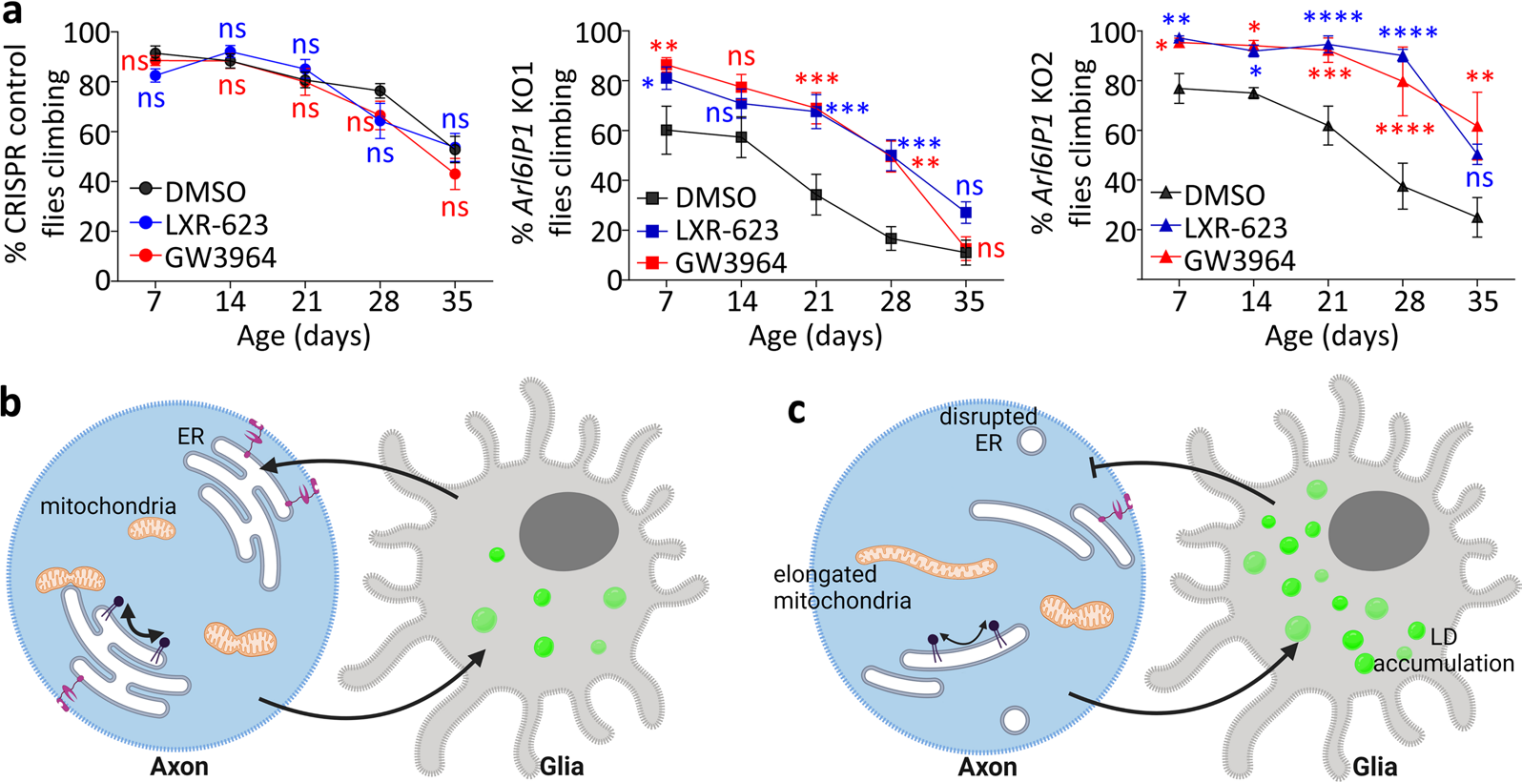
Treatment with LXR-receptor agonists improves locomotor behaviour in *Arl6IP1* KO models of SGP61. **a** Graph represent mean ± SEM percentage of CRISPR Control or *Arl6IP1* KO flies that reach the top of the vial within 15 s. *n* = 10–16 independent experiments per genotype. Statistics consists of two-way ANOVA and Bonferroni’s multiple comparisons tests. **b** and **c** Proposed model for ARL6IP1 function in lipid homeostasis within axon bundles**.** **b** In wildtype systems, lipids required by neurons are stored in glia and then transferred into neurons when needed. The ER functions to regulate lipid homeostasis via ER-localised lipid transporters and lipid metabolising enzymes. **b** Loss of function Arl6IP1 mutations, similar to those known to cause HSP in patients, disrupt ER organisation and lipid homeostasis resulting in LD accumulation within adjacent glia. Lipid disruption contributes to disease pathogenesis as restoration of lipid balance by treatment with LXR-agonists, which enhance cholesterol efflux from glia, reduces neurodegeneration in our fly model of HSP

Having shown that we could pharmacologically restore LD organisation in *Arl6IP1* KO flies, we investigated whether lipid disruption is relevant for HSP-associated neurodegeneration. We therefore tested whether restoring lipid homeostasis using LXR-agonists has any effect on the neurodegeneration phenotypes caused by loss of Arl6IP1. Both LXR agonists caused a marked improvement in ER and mitochondrial organisation within long motor neurons. While Rtnl1::YFP staining remains less intense in *Arl6IP1* KO lines compared to controls, there is a significant increase in continuity of this smooth ER marker (Fig. 8a, b). Mitochondrial morphology within long motor neuron axons of *Arl6IP1* KO flies is also restored to that seen in control flies by chronic treatment with either LXR-agonist (Fig. 8c–e). Finally, treatment with either LXR agonist results in a marked improvement in the locomotor scores in *Arl6IP1* KO flies (Fig. 9a). Taken together, these data indicate that ARL6IP1-mediated regulation of lipid homeostasis contributes to long motor neuron maintenance in this in vivo model of HSP.

## Discussion

Mutations in genes encoding ER-shaping proteins are a common cause of the neurodegenerative disorder HSP. To investigate the mechanisms underpinning neurodegeneration due to loss of the ER-shaping protein *ARL6IP1*, mutations in which cause an autosomal-recessive form of HSP (SPG61), we generated novel models of disease by CRISPR/Cas9-mediated knockout of *ARL6IP1* in *Drosophila* and a human cell line. These models should be more representative of SPG61 than previous RNAi models [31] as SPG61 patients present with homozygous mutations believed to confer complete loss of protein [12, 54, 90]. *Arl6IP1* KO flies display progressive locomotor deficits in the absence of reduced survival, therefore recapitulating some, but not all, symptomatology associated with SPG61. We show that very long motor neuron axons are particularly susceptible to loss of Arl6IP1 as tubular ER is disrupted in long, but not short, axon terminals. Similarly, mitochondrial elongation, previously linked to disrupted mitochondrial fission [29], is evident in long, but not short motor neuron axons. Unexpectedly, our findings in both *Drosophila* and human model systems indicate that ARL6IP1 has a highly conserved role in the regulation of lipid homeostasis with ARL6IP1 KO models presenting with disrupted lipid droplet organisation. In particular, we have identified a novel physical and functional interaction between ARL6IP1 and the phosopholipid transport protein ORP8. Within axon bundles, loss of Arl6IP1 in neurons triggers accumulation of LDs in the glia surrounding neuronal axons. This is a cell non-autonomous effect as loss of Arl6IP1 in glia does not trigger LD accumulation or progressive locomotor defects. Finally, we show that treatment with LXR-agonists prevents LD enlargement within glia, restores tubular ER and mitochondrial organisation within long axons, and improves locomotor ability in our *Drosophila* model of SPG61.

A key finding of this work is that ARL6IP1 has highly conserved, and functionally relevant, roles in tubular ER organisation and lipid homeostasis. Within human U-2 OS cells, ARL6IP1 localises throughout the tubular ER, extending to the ER tips, which mediate ER-PM contacts [30], consistent with findings in other mammalians cell models [24]. Analysis of protein–protein interactors reveals that almost half of identified ARL6IP1 interactors are ER-localised and ER-shaping proteins, including RTN3 and REEP5 which function to regulate ER organisation [11, 37, 76, 88, 96]. Within long motor neurons in *Drosophila*, loss of Arl6IP1 results in disruption of the axonal tubular ER network. Given that is it the longest motor neuron axons that particularly degenerate in HSP, a reasonable hypothesis is that longer axons are more susceptible to changes that impair the diameter, dynamics, branching or continuity of tubular ER [56, 85, 99]. Our analysis of ARL6IP1-interacting proteins also highlighted a subset of proteins that localise to ER-PM contacts and regulate phosopholipid availability including: VAPA [42], extended-synaptotagmin 1 (E-SYT1) [6], INPP5K [24], RTN3 [11], and ORP8 [18, 33]. Of these, we show that the ARL6IP1-ORP8 interaction is functionally conserved in *Drosophila*. Moreover, loss of ARL6IP1 in both human cell and *Drosophila* model systems results in defective LD organisation. Interestingly, the nature of the LD disruption appears to differ according to the ARL6IP1 KO cell type with reduced LDs in U-2 OS cells, no change in LDs in axonal neurons and an increase in LD accumulation in axonal glia. These findings may be explained by well documented differences in cell-type specific intracellular lipid content between cultured cells, primary cells, and even between discrete brain regions [28, 82]. Nonetheless, our findings suggest that ARL6IP1 has a highly conserved role in the regulation of lipid homeostasis.

Our findings also suggest that disrupted lipid homeostasis caused by loss of ARL6IP1 is pathophysiologically relevant for neurodegeneration in HSP. This supports research which demonstrates both direct and indirect disruption of lipid homeostasis in HSP (reviewed in [19, 84]). Mutations in several genes encoding enzymes which regulate lipid metabolism cause HSP. These include the ER-LD tether Seipin (causing SPG17) [94], as well as several genes encoding enzymes which regulate lipid metabolism, including DDHD1 and DDHD2 (causing SPG28 and SPG54 respectively) [8, 73], fatty acid 2-hydroxylase (FA2H; causing SPG35) [21], patatin like phospholipase domain containing 6 (PNPLA6; causing SPG39) [63] and carnitine palmitoyl-transferase (CPT1C; causing SPG73) [67]. Disrupted lipid content and LD defects are common in animal models and patient samples associated with these mutations. Interestingly, LD defects have also been detected in models of HSP which are not known to directly function in lipid metabolism or LD biogenesis. Mutations in genes encoding the ER-shaping proteins Spastin, Atlastin-1, and REEP1 have all been shown to disrupt LD formation [44, 57, 66]. Whether this occurs as a result of the defective tubular ER network organization that loss of these proteins causes, or through specific roles for these proteins in LD organization, is not currently understood. It was recently reported that treatment with the LXR-agonist GW3965 restores lipid balance in neurons differentiated from SPG3A patient-derived pluripotent stem cells [50]. This treatment reduced axonal swellings, suggesting that LXR-agonists might offer potential as a novel therapeutic strategy for HSP. However, given that ER-shaping protein-mediated LD disruption and rescue by LXR-agonists have largely been investigated in vitro or in adipose tissue in vivo, the relevance of these to axonopathy in long motor neuron was not known. Here, we present the first evidence that LXR-agonists can be used to restore lipid homeostasis and rescue neurodegeneration in an in vivo model of HSP.

Several questions still remain, particularly concerning the mechanism by which loss of ARL6IP1 in neurons causes LD accumulation in glia, and how is this contributing to axonopathy in neurons? Neurons themselves do not store significant amounts of lipids. Instead, glia within the nervous system store lipids in LDs which get metabolised and the metabolites are transferred to neurons as needed (Fig. 9b) [4]. Neuronal axons require a reliable supply of lipids from glia to maintain functions including membrane plasticity (e.g. tubular ER and mitochondrial dynamics), endo- and exocytosis, as well as lipid raft formation. Disruption of neuronal phospholipid availability, either by loss of glia-to-neuron lipid transporters [25, 92] or loss of enzymes regulating phospholipid metabolism [55, 93], leads to neuronal defects and is associated with neuronal dysfunction in humans, underpinning the importance of this pathway in neuronal maintenance.

Given that ARL6IP1 interacts with several proteins involved in the regulation of phospholipids within cells, one model that could explain the findings presented here is that loss of ARL6IP1 reduces lipid availability within neurons perhaps through altered tubular ER organisation, disrupted phospholipid transfer into neurons (e,g, via ORP8), and/or impaired phospholipid metabolism (e.g. via INPP5K, MBOAT7) (Fig. 9c). Reduced lipid homeostasis within neurons can disrupt membrane plasticity and could contribute to disrupted tubular ER and mitochondrial organisation, as observed within long motor neuron axons in *Arl6IP1* KO flies. By increasing the concentration of lipids at the neuro-glia interface, LXR-agonists may promote phospholipid availability within neurons, thereby helping to restore tubular ER and mitochondrial organisation. An alternative model is that glia are acting to resolve neuronal stress induced by loss of ARL6IP1. Neuronal stressors, such as defects in the mitochondrial respiratory chain, elevate ROS and lead to the increased production of lipids. As lipids cannot be stored within neurons, they are transferred to glia where they accumulate as LDs [45]. When elevated neuronal ROS underpins glial LD accumulation, treatment with antioxidants, such as AD4, to decrease ROS production in neurons reduces LD accumulation in glia and delays neurodegeneration [3, 45, 71]. However, in this study, treatment with AD4 did not reliably reduce LD accumulation in *Arl6IP1* KO glia, which is inconsistent with this model and suggests that neurodegeneration associated with loss of ARL6IP1 is not a result of elevated ROS production in neurons.

Our discovery that loss of the ER-shaping protein ARL6IP1 impairs lipid droplet organisation leading to LD accumulation in glia surrounding long motor neuron axons adds to the growing body of research linking lipid homeostasis disruption and HSP. Moreover, our results are the first to show that drug treatments to restore lipid homeostasis modify HSP-associated neurodegenerative phenotypes in vivo. While future studies will be required to provide a greater understanding of the mechanisms by which loss of ER-shaping proteins impacts cross-talk between neuronal axons and glia, this work suggests that restoration of lipid homeostasis could be an effective therapeutic strategy for reducing neurodegeneration underpinning HSP.

## Supplementary Information

**Additional file 1:** Donor DNA sequences used for CRISPR-Cas9 gene editing.

**Additional file 2:** Supplementary Figs. 1–6.

**Additional file 3:** Supplementary Table 1. List of proteins identified as likely interactors of human ARL6IP1. This list of proteins was generated from the proteins pulled down with ARL6IP1-FLAG. Those included in this list had a spectral intensity abundance ratio (Arl6IP1-FLAG:Control) greater than 1.2 and were not identified in the CRAPome as non-specific binding partners within U-2 OS cells incubated with FLAG M2 affinity beads [48]. GO analysis was conducted to determine the localisation of ARL6IP1 binding partners and shown are proteins identified to have significant enrichment within ER, ER membrane or ER tubular network as determined by D = DAVID, P = Panther, FA = FuncAssociate, E = Enrichr. Proteins in bold were validated as ARL6IP1 interactors by independent co-immunoprecipitation and co-localisation experiments in this study. PSM: peptide spectrum matches.
