# Supplementary material for: Liver X receptor-agonist treatment rescues degeneration in a Drosophila model of hereditary spastic paraplegia
Source: Acta Neuropathol Commun. 2022 Mar 28;10:40. doi: 10.1186/s40478-022-01343-6 (PMC8961908; doi:10.1186/s40478-022-01343-6)
Supplement: Supplementary file 1 — Additional file 1: Donor DNA sequences used for CRISPR-Cas9 gene editing. [file 40478_2022_1343_MOESM1_ESM.pdf]

***Drosophila* Arl6IP1 Knockout ssDonor DNA sequence:**

5’–ATAATGATTCCTCATTTTCCGGCATTTTCCAGAAACGAGCACTTAACAAATTAAAGCACGACTGATAATAGT TGGAGCCTTTCCGGACAGCGATTGTTGGAGCCGTACGGCGTGCTAACCTGGGAGAAGCAGTACTACGCCGGA GTGGTGTTTGGCGTCATCAGCT–3’

***Drosophila* Arl6IP1 Knockin ssDonor DNA for endogenous tagging sequence:**

5’–CAAAGGGCCTCGAAGATTATTAACGAGAAGATCCAGTGCGGCAAAAGGAAATTGCAAGGAGGCGGTAG CGACTATAAAGATCACGACGGTGATTACAAAGATCATGATATTGACTACAAAGACGACGACGATAAGTGATA AAGGAAAGCAACCAGATAAACTCATCACCATTTGACCAAAAATATAAA ACATGCCTAAGT–3’

***ARL6IP1* specific Homology arms for endogenous tagging sequence:**

ARL6IP1-HomologyArm1- Forward: 5’-ACCATATGAGCGCTGCAGATCTGAGCTCGTACATTGGAATGGCCA AGAGGGGTACTAAAAATACAAAAATTAGCCAGGCGTGGCG-3’

ARL6IP1-HomologyArm1- Reverse: 5’-CTGCTGGCGCCGGCGCCGGATCCTTCGTTTTTCTTTTCTTTTTGTTT GAGAAGTTTGTTTATCTCtCTCTTGGCCATTCCAATGTAC-3’

ARL6IP1-HomologyArm2-Forward: 5’- AGCATACATTATACGAAGTTATGAATTCTTCATCTGCTTTAATCAGT GTGATTAATGC-3’

ARL6IP1-HomologyArm2-Reverse: 5’-TTTGCTGGCCTTTTGCTCACATGTCCTACTGTATACTATAGCTAATGT CAGC-3’

Double stranded donor DNA vector sequence for endogenously tagging Human ARL6IP1 (neomycin):

5’–TCGCGCGTTTCGGTGATGACGGTGAAAACCTCTGACACATGCAGCTCCCGGAGACGGTCACAGCTTGTCT GTAAGCGGATGCCGGGAGCAGACAAGCCCGTCAGGGCGCGTCAGCGGGTGTTGGCGGGTGTCGGGGCTGG CTTAACTATGCGGCATCAGAGCAGATTGTACTGAGAGTGCACCATATGAGCGCTGCAGATCTGAGCTCGTACA TTGGAATGGCCAAGAGGGGTACTAAAAATACAAAAATTAGCCAGGCGTGGCGGCAGGCACCTGTAATCCCAG CTACTCTACTTGGTAGGGTGAGGCAGGAGAATCGCTTGAACCTGGGAGGTGGAGGTTGCAGTGAACCGAGA TTGTGCCATTGCACTCCAGCCTGGGCAAAAAGAGTGAGACTTGTCGCTGGGTGTGGTGGCTCACGCCCGTAAT CCCAGCACTCTGGGAGGCTGAAGCAGGCGGATCACCTGAGATTGGGAGTTCGAGACCAGCCTGACTAACATG GAGAAACCCCATCTCTACTAAAAATACAAAATTAGTCAGGCGTAGTGGCGCATGCCTGTAATCCCAGCTACTT GGGTAGCTGAGGCAGGAGAATCGCTTGAACCTGGGAGGCGGAGGTTGCGGTGAGCCAAGATTGCGCCATTG CACTCCAGCCTGGGCAACAAAAGTGAAACTTTGTCTCAAAAAAAAAAAAAAAAAGTAATAACCTTTAGCAGGT TAATTGCTGCCTCCTTAATGTTGGGTTTTTTTTTATTTTAAGTGACTTCCTTACTATTGCTTCCTGGACTAAACCA ACATGGAATCATTTTGAAGTACATTGGAATGGCCAAGAGAGAGATAAACAAACTTCTCAAACAAAAAGAAAA GAAAAACGAAGGATCCGGCGCCGGCGCCAGCAGCGGTGGAGGTGGATCTGACTCTAAAGAGAAGTCAGCTT GTCCTAAGGACCCTGCCAAGCCACCTGCGAAGGCCCAAGTAGTCGGTTGGCCACCCGTGCGCAGCTATAGGA AGAACGTGATGGTGTCCTGTCAGAAGTCTAGCGGGGGGCCCGAGGCTTCCGGTGGCGGCTCTGGCGGATCT GGTGGAGGCGGTGCTAGCTCCGCCGGCGACTACAAGGACCACGACGGCGATTATAAGGATCACGACATCGA CTACAAAGACGACGATGACAAGGGCGCCGGAGGCGGTTCTGGAGGCGGTTCTCCCGACAGGGTGAGGGCCG TGAGCCACTGGAGCAGCTGAAGGCTGTGCCTTCTAGTTGCCAGCCATCTGTTGTTTGCCCCTCCCCCGTGCCTT CCTTGACCCTGGAAGGTGCCACTCCCACTGTCCTTTCCTAATAAAATGAGGAAATTGCATCGCATTGTCTGAGT

AGGTGTCATTCTATTCTGGGGGGTGGGGTGGGGCAGGACAGCAAGGGGGAGGATTGGGAAGACAATAGCA
GGCATGCTGGGGATGCGGTGGGCTCTATGGGTCGACATAACTTCGTATAGCATACATTATACGAAGTTATGAT
CTGCGATCGCTCCGGTGCCCGTCAGTGGGCAGAGCGCACATCGCCCACAGTCCCCGAGAAGTTGGGGGGAG
GGGTCGGCAATTGAACCGGTGCCTAGAGAAGGTGGCGCGGGGTAAACTGGGAAAGTGATGTCGTGTACTGG
CTCCGCCTTTTTCCCGAGGGTGGGGGAGAACCGTATATAAGTGCAGTAGTCGCCGTGAACGTTCTTTTTCGCA
ACGGGTTTGCCGCCAGAACACAGCTGAAGCTTCGAGGGGCTCGCATCTCTCCTTCACGCGCCCGCCGCCCTAC
CTGAGGCCGCCATCCACGCCGGTTGAGTCGCGTTCTGCCGCCTCCCGCCTGTGGTGCCTCCTGAACTGCGTCC
GCCGTCTAGGTAAGTTTAAAGCTCAGGTCGAGACCGGGCCTTTGTCCGGCGCTCCCTTGGAGCCTACCTAGAC
TCAGCCGGCTCTCCACGCTTTGCCTGACCCTGCTTGCTCAACTCTACGTCTTTGTTTCGTTTTCTGTTCTGCGCCG
TTACAGATCCAAGCTGTGACCGGCGCCTACCTCGAGATTGAACAAGATGGATTGCACGCAGGTTCTCCGGCCG
CTTGGGTGGAGAGGCTATTCGGCTATGACTGGGCACAACAGACAATCGGCTGCTCTGATGCCGCCGTGTTCC
GGCTGTCAGCGCAGGGGCGCCCGGTTCTTTTTGTCAAGACCGACCTGTCCGGTGCCCTGAATGAACTGCAGG
ACGAGGCAGCGCGGCTATCGTGGCTGGCCACGACGGGCGTTCCTTGCGCAGCTGTGCTCGACGTTGTCACTG
AAGCGGGAAGGGACTGGCTGCTATTGGGCGAAGTGCCGGGGCAGGATCTCCTGTCATCTCACCTTGCTCCTG
CCGAGAAAGTATCCATCATGGCTGATGCAATGCGGCGGCTGCATACGCTTGATCCGGCTACCTGCCCATTCGA
CCACCAAGCGAAACATCGCATCGAGCGAGCACGTACTCGGATGGAAGCCGGTCTTGTCGATCAGGATGATCT
GGACGAAGAGCATCAGGGGCTCGCGCCAGCCGAACTGTTCGCCAGGCTCAAGGCGCGCATGCCCGACGGCG
AGGATCTCGTCGTGACCCATGGCGATGCCTGCTTGCCGAATATCATGGTGGAAAATGGCCGCTTTTCTGGATT
CATCGACTGTGGCCGGCTGGGTGTGGCGGACCGCTATCAGGACATAGCGTTGGCTACCCGTGATATTGCTGA
AGAGCTTGGCGGCGAATGGGCTGACCGCTTCCTCGTGCTTTACGGTATCGCCGCTCCCGATTCGCAGCGCATC
GCCTTCTATCGCCTTCTTGACGAGTTCTTCTAAACGCGTTAAAATATCTTTATTTTCATTACATCTGTGTGTTGGT
TTTTTGTGTGAATCGATAGTACTAACATACGCTCTCCATCAAAACAAAACGAAACAAAACAAACTAGCAAAATA
GGCTGTCCCCAGTGCAAGTGCAGGTGCCAGAACATTTCTCTCTCGCGGCATGGACGAGTAAGGTACCATAACT
TCGTATAGCATACATTATACGAAGTTATGAATTCTTCATCTGCTTTAATCAGTGTGATTAATGCAGCACCCATTG
CCCCGGGAACCGTTTCTGCTGTACTATCTGGATACTAAAATGTTACGGAAGTAGCTCTTTGTTCTCCCTCACTCT
GCCCTTAGTTAATAGAAATTCAGACTCGCCAAGTAAGGCTTCGTGCATAGTGTCTTCATGTCGCGTATAGTTGA
GCGCGTTCTTAGCAGTTGGCTTCATGGACAACTCATTAGTGTTTTGACTTTTCTTACCCAGCGTTAATTGAATTC
TTGCTTTTAGACAACTTCCTTTTTGTAGTGGTGAACCTTGCCCTTTAGTACAGTTCAAGTGAATCTGGATAATTG
TTCATCTTTGCTTTAGCTTAGATACCATGTAGTGGTCTGTGGCTACAGGAAGCTGGTTCTGTCTGCTTCCACAG
TCTGCTTAAAAAACTGTCTGACTTCGTGAATATAGAGACCAAGTTTACCACTTCTGATGAAGAGACCAATTAAG
ATTCATTCCTCATTCTGTTTCTTTCCAGTGGGAGAAGAGTCCCCATGAAATAAGATGAAACTGATTCCATGCAC
TAGTACATGTAGGCTTCTCCCTTGTGCAAAGCTTAGCAATTTGTAGGAAACTTTGATCTTTTTGTCCAAGAAAA
GGAATGTCTGACAGGCTTAAGCTTTCGTCCCCTTGCACTTAGACTCGAAGTTAGTAAATCCTTAAAGGCTTTTT
AATAGCAGACTTCCAAAAGATTGCATTTAGGATTTCTAGCATGCTTTTAATTTCAGATTTTCAGCTGACATTAGC
TATAGTATACAGTAGGACATGTGAGCAAAAGGCCAGCAAAAGGCCAGGAACCGTAAAAAGGCCGCGTTGCT
GGCGTTTTTCCATAGGCTCCGCCCCCCTGACGAGCATCACAAAAATCGACGCTCAAGTCAGAGGTGGCGAAAC
CCGACAGGACTATAAAGATACCAGGCGTTTCCCCCTGGAAGCTCCCTCGTGCGCTCTCCTGTTCCGACCCTGCC
GCTTACCGGATACCTGTCCGCCTTTCTCCCTTCGGGAAGCGTGGCGCTTTCTCATAGCTCACGCTGTAGGTATC
TCAGTTCGGTGTAGGTCGTTCGCTCCAAGCTGGGCTGTGTGCACGAACCCCCCGTTCAGCCCGACCGCTGCGC
CTTATCCGGTAACTATCGTCTTGAGTCCAACCCGGTAAGACACGACTTATCGCCACTGGCAGCAGCCACTGGT
AACAGGATTAGCAGAGCGAGGTATGTAGGCGGTGCTACAGAGTTCTTGAAGTGGTGGCCTAACTACGGCTAC
ACTAGAAGGACAGTATTTGGTATCTGCGCTCTGCTGAAGCCAGTTACCTTCGGAAAAAGAGTTGGTAGCTCTT
GATCCGGCAAACAAACCACCGCTGGTAGCGGTGGTTTTTTTGTTTGCAAGCAGCAGATTACGCGCAGAAAAA
AAGGATCTCAAGAAGATCCTTTGATCTTTTCTACGGGGTCTGACGCTCAGTGGAACGAAAACTCACGTTAAGG
GATTTTGGTCATGAGATTATCAAAAAGGATCTTCACCTAGATCCTTTTAAATTAAAAATGAAGTTTTAAATCAA
TCTAAAGTATATATGAGTAAACTTGGTCTGACAGTTACCAATGCTTAATCAGTGAGGCACCTATCTCAGCGATC
TGTCTATTTCGTTCATCCATAGTTGCCTGACTCCCCGTCGTGTAGATAACTACGATACGGGAGGGCTTACCATC
TGGCCCCAGTGCTGCAATGATACCGCGAGACCCACGCTCACCGGCTCCAGATTTATCAGCAATAAACCAGCCA

GCCGGAAGGGCCGAGCGCAGAAGTGGTCCTGCAACTTTATCCGCCTCCATCCAGTCTATTAATTGTTGCCGGG
AAGCTAGAGTAAGTAGTTCGCCAGTTAATAGTTTGCGCAACGTTGTTGCCATTGCTACAGGCATCGTGGTGTC
ACGCTCGTCGTTTGGTATGGCTTCATTCAGCTCCGGTTCCCAACGATCAAGGCGAGTTACATGATCCCCCATGT
TGTGCAAAAAAGCGGTTAGCTCCTTCGGTCCTCCGATCGTTGTCAGAAGTAAGTTGGCCGCAGTGTTATCACT
CATGGTTATGGCAGCACTGCATAATTCTCTTACTGTCATGCCATCCGTAAGATGCTTTTCTGTGACTGGTGAGT
ACTCAACCAAGTCATTCTGAGAATAGTGTATGCGGCGACCGAGTTGCTCTTGCCCGGCGTCAATACGGGATAA
TACCGCGCCACATAGCAGAACTTTAAAAGTGCTCATCATTGGAAAACGTTCTTCGGGGCGAAAACTCTCAAGG
ATCTTACCGCTGTTGAGATCCAGTTCGATGTAACCCACTCGTGCACCCAACTGATCTTCAGCATCTTTTACTTTC
ACCAGCGTTTCTGGGTGAGCAAAAACAGGAAGGCAAAATGCCGCAAAAAAGGGAATAAGGGCGACACGGAA
ATGTTGAATACTCATACTCTTCCTTTTTCAATATTATTGAAGCATTTATCAGGGTTATTGTCTCATGAGCGGATA
CATATTTGAATGTATTTAGAAAAATAAACAAATAGGGGTTCCGCGCACATTTCCCCGAAAAGTGCCACCTGAC
GTCTAAGAAACCATTATTATCATGACATTAACCTATAAAAATAGGCGTATCACGAGGCCCTTTCGTC–3’

Double stranded donor DNA vector sequence for endogenously tagging Human ARL6IP1 (puromycin):

5’–TCGCGCGTTTCGGTGATGACGGTGAAAACCTCTGACACATGCAGCTCCCGGAGACGGTCACAGCTTGTCT
GTAAGCGGATGCCGGGAGCAGACAAGCCCGTCAGGGCGCGTCAGCGGGTGTTGGCGGGTGTCGGGGCTGG
CTTAACTATGCGGCATCAGAGCAGATTGTACTGAGAGTGCACCATATGAGCGCTGCAGATCTGAGCTCGTACA
TTGGAATGGCCAAGAGGGGTACTAAAAATACAAAAATTAGCCAGGCGTGGCGGCAGGCACCTGTAATCCCAG
CTACTCTACTTGGTAGGGTGAGGCAGGAGAATCGCTTGAACCTGGGAGGTGGAGGTTGCAGTGAACCGAGA
TTGTGCCATTGCACTCCAGCCTGGGCAAAAAGAGTGAGACTTGTCGCTGGGTGTGGTGGCTCACGCCCGTAAT
CCCAGCACTCTGGGAGGCTGAAGCAGGCGGATCACCTGAGATTGGGAGTTCGAGACCAGCCTGACTAACATG
GAGAAACCCCATCTCTACTAAAAATACAAAATTAGTCAGGCGTAGTGGCGCATGCCTGTAATCCCAGCTACTT
GGGTAGCTGAGGCAGGAGAATCGCTTGAACCTGGGAGGCGGAGGTTGCGGTGAGCCAAGATTGCGCCATTG
CACTCCAGCCTGGGCAACAAAAGTGAAACTTTGTCTCAAAAAAAAAAAAAAAAAAGTAATAACCTTTAGCAGGT
TAATTGCTGCCTCCTTAATGTTGGGTTTTTTTTTATTTTAAGTGACTTCCTTACTATTGCTTCCTGGACTAAACCA
ACATGGAATCATTTTGAAGTACATTGGAATGGCCAAGAGAGAGATAAACAAACTTCTCAAACAAAAAGAAAA
GAAAAACGAAGGATCCGGCGCCGGCGCCAGCAGCGGTGGAGGTGGATCTGACTCTAAAGAGAAGTCAGCTT
GTCCTAAGGACCCTGCCAAGCCACCTGCGAAGGCCCAAGTAGTCGGTTGGCCACCCGTGCGCAGCTATAGGA
AGAACGTGATGGTGTCCTGTCAGAAGTCTAGCGGGGGGCCCGAGGCTTCCGGTGGCGGCTCTGGCGGATCT
GGTGGAGGCGGTGCTAGCTCCGCCGGCGACTACAAGGACCACGACGGCGATTATAAGGATCACGACATCGA
CTACAAAGACGACGATGACAAGGGCGCCGGAGGCGGTTCTGGAGGCGGTTCTCCCGACAGGGTGAGGGCCG
TGAGCCACTGGAGCAGCTGAAGGCTGTGCCTTCTAGTTGCCAGCCATCTGTTGTTTGCCCCTCCCCCGTGCCTT
CCTTGACCCTGGAAGGTGCCACTCCCACTGTCCTTTCCTAATAAAATGAGGAAATTGCATCGCATTGTCTGAGT
AGGTGTCATTCTATTCTGGGGGGTGGGGTGGGGCAGGACAGCAAGGGGGAGGATTGGGAAGACAATAGCA
GGCATGCTGGGGATGCGGTGGGCTCTATGGGTCGACATAACTTCGTATAGCATACATTATACGAAGTTATGAT
CTGCGATCGCTCCGGTGCCCGTCAGTGGGCAGAGCGCACATCGCCCACAGTCCCCGAGAAGTTGGGGGGAG
GGGTCGGCAATTGAACCGGTGCCTAGAGAAGGTGGCGCGGGGTAAACTGGGAAAGTGATGTCGTGTACTGG
CTCCGCCTTTTTCCCGAGGGTGGGGGAGAACCGTATATAAGTGCAGTAGTCGCCGTGAACGTTCTTTTTCGCA
ACGGGTTTGCCGCCAGAACACAGCTGAAGCTTCGAGGGGCTCGCATCTCTCCTTCACGCGCCCGCCGCCCTAC
CTGAGGCCGCCATCCACGCCGGTTGAGTCGCGTTCTGCCGCCTCCCGCCTGTGGTGCCTCCTGAACTGCGTCC
GCCGTCTAGGTAAGTTTAAAGCTCAGGTCGAGACCGGGCCTTTGTCCGGCGCTCCCTTGGAGCCTACCTAGAC
TCAGCCGGCTCTCCACGCTTTGCCTGACCCTGCTTGCTCAACTCTACGTCTTTGTTTCGTTTTCTGTTCTGCGCCG
TTACAGATCCAAGCTGTGACCGGCGCCTACCTCGAGATGACCGAGTACAAGCCCACAGTGCGGCTGGCCACC
AGGGACGATGTGCCTAGAGCTGTGCGGACACTGGCCGCTGCCTTCGCCGATTACCCTGCCACCAGACACACC
GTGGACCCCGACAGACACATCGAGAGAGTGACCGAGCTGCAGGAACTGTTTCTGACCAGAGTGGGCCTGGA

CATCGGCAAAGTGTGGGTGGCCGATGATGGCGCCGCTGTGGCTGTGTGGACAACCCCTGAGTCTGTGGAAGC
CGGCGCTGTGTTCGCCGAGATCGGACCTAGAATGGCCGAGCTGAGCGGCTCTAGACTGGCTGCCCAGCAGCA
GATGGAAGGCCTGCTGGCCCCCCACAGACCTAAAGAGCCTGCCTGGTTTCTGGCCACCGTGGGCGTGTCACCT
GACCACCAGGGCAAGGGACTGGGATCTGCTGTGGTGCTGCCTGGCGTGGAAGCTGCTGAAAGGGCTGGCGT
GCCCGCCTTCCTGGAAACAAGCGCCCCCAGAAACCTGCCCTTCTACGAGAGACTGGGCTTCACCGTGACCGCC
GACGTGGAAGTGCCTGAGGGCCCTAGAACCTGGTGCATGACCAGAAAGCCTGGCGCCTGAACGCGTTAAAAT
ATCTTTATTTTCATTACATCTGTGTGTTGGTTTTTTGTGTGAATCGATAGTACTAACATACGCTCTCCATCAAAAC
AAAACGAAACAAAACAAACTAGCAAAATAGGCTGTCCCCAGTGCAAGTGCAGGTGCCAGAACATTTCTCTCTC
GCGGCATGGACGAGTAAGGTACCATAACTTCGTATAGCATACATTATACGAAGTTATGAATTCTTCATCTGCTT
TAATCAGTGTGATTAATGCAGCACCCATTGCCCCGGGAACCGTTTCTGCTGTACTATCTGGATACTAAAATGTT
ACGGAAGTAGCTCTTTGTTCTCCCTCACTCTGCCCTTAGTTAATAGAAATTCAGACTCGCCAAGTAAGGCTTCG
TGCATAGTGTCTTCATGTCGCGTATAGTTGAGCGCGTTCTTAGCAGTTGGCTTCATGGACAACTCATTAGTGTT
TTGACTTTTCTTACCCAGCGTTAATTGAATTCTTGCTTTTAGACAACTTCCTTTTTGTAGTGGTGAACCTTGCCCT
TTAGTACAGTTCAAGTGAATCTGGATAATTGTTCATCTTTGCTTTAGCTTAGATACCATGTAGTGGTCTGTGGC
TACAGGAAGCTGGTTCTGTCTGCTTCCACAGTCTGCTTAAAAAACTGTCTGACTTCGTGAATATAGAGACCAAG
TTTACCACTTCTGATGAAGAGACCAATTAAGATTCATTCCTCATTCTGTTTCTTTCCAGTGGGAGAAGAGTCCCC
ATGAAATAAGATGAAACTGATTCCATGCACTAGTACATGTAGGCTTCTCCCTTGTGCAAAGCTTAGCAATTTGT
AGGAAACTTTGATCTTTTTGTCCAAGAAAAGGAATGTCTGACAGGCTTAAGCTTTCGTCCCCTTGCACTTAGAC
TCGAAGTTAGTAAATCCTTAAAGGCTTTTTAATAGCAGACTTCCAAAAGATTGCATTTAGGATTTCTAGCATGC
TTTTAATTTCAGATTTTCAGCTGACATTAGCTATAGTATACAGTAGGACATGTGAGCAAAAGGCCAGCAAAAG
GCCAGGAACCGTAAAAAGGCCGCGTTGCTGGCGTTTTTCCATAGGCTCCGCCCCCCTGACGAGCATCACAAAA
ATCGACGCTCAAGTCAGAGGTGGCGAAACCCGACAGGACTATAAAGATACCAGGCGTTTCCCCCTGGAAGCT
CCCTCGTGCGCTCTCCTGTTCCGACCCTGCCGCTTACCGGATACCTGTCCGCCTTTCTCCCTTCGGGAAGCGTG
GCGCTTTCTCATAGCTCACGCTGTAGGTATCTCAGTTCGGTGTAGGTCGTTCGCTCCAAGCTGGGCTGTGTGCA
CGAACCCCCCGTTCAGCCCGACCGCTGCGCCTTATCCGGTAACTATCGTCTTGAGTCCAACCCGGTAAGACAC
GACTTATCGCCACTGGCAGCAGCCACTGGTAACAGGATTAGCAGAGCGAGGTATGTAGGCGGTGCTACAGAG
TTCTTGAAGTGGTGGCCTAACTACGGCTACACTAGAAGGACAGTATTTGGTATCTGCGCTCTGCTGAAGCCAG
TTACCTTCGGAAAAAGAGTTGGTAGCTCTTGATCCGGCAAACAAACCACCGCTGGTAGCGGTGGTTTTTTTGT
TTGCAAGCAGCAGATTACGCGCAGAAAAAAAGGATCTCAAGAAGATCCTTTGATCTTTTCTACGGGGTCTGAC
GCTCAGTGGAACGAAAACTCACGTTAAGGGATTTTGGTCATGAGATTATCAAAAAGGATCTTCACCTAGATCC
TTTTAAATTAAAAATGAAGTTTTAAATCAATCTAAAGTATATATGAGTAAACTTGGTCTGACAGTTACCAATGC
TTAATCAGTGAGGCACCTATCTCAGCGATCTGTCTATTTCGTTCATCCATAGTTGCCTGACTCCCCGTCGTGTAG
ATAACTACGATACGGGAGGGCTTACCATCTGGCCCCAGTGCTGCAATGATACCGCGAGACCCACGCTCACCG
GCTCCAGATTTATCAGCAATAAACCAGCCAGCCGGAAGGGCCGAGCGCAGAAGTGGTCCTGCAACTTTATCC
GCCTCCATCCAGTCTATTAATTGTTGCCGGGAAGCTAGAGTAAGTAGTTCGCCAGTTAATAGTTTGCGCAACGT
TGTTGCCATTGCTACAGGCATCGTGGTGTCACGCTCGTCGTTTGGTATGGCTTCATTCAGCTCCGGTTCCCAAC
GATCAAGGCGAGTTACATGATCCCCCATGTTGTGCAAAAAAGCGGTTAGCTCCTTCGGTCCTCCGATCGTTGTC
AGAAGTAAGTTGGCCGCAGTGTTATCACTCATGGTTATGGCAGCACTGCATAATTCTCTTACTGTCATGCCATC
CGTAAGATGCTTTTCTGTGACTGGTGAGTACTCAACCAAGTCATTCTGAGAATAGTGTATGCGGCGACCGAGT
TGCTCTTGCCCGGCGTCAATACGGGATAATACCGCGCCACATAGCAGAACTTTAAAAGTGCTCATCATTGGAA
AACGTTCTTCGGGGCGAAAACTCTCAAGGATCTTACCGCTGTTGAGATCCAGTTCGATGTAACCCACTCGTGC
ACCCAACTGATCTTCAGCATCTTTTACTTTCACCAGCGTTTCTGGGTGAGCAAAAACAGGAAGGCAAAATGCC
GCAAAAAAGGGAATAAGGGCGACACGGAAATGTTGAATACTCATACTCTTCCTTTTTCAATATTATTGAAGCA
TTTATCAGGGTTATTGTCTCATGAGCGGATACATATTTGAATGTATTTAGAAAAATAAACAAATAGGGGTTCCG
CGCACATTTCCCCGAAAAGTGCCACCTGACGTCTAAGAAACCATTATTATCATGACATTAACCTATAAAAATAG
GCGTATCACGAGGCCCTTTCGTC–3’
