# Supplementary material for: Liver X receptor-agonist treatment rescues degeneration in a Drosophila model of hereditary spastic paraplegia
Source: Acta Neuropathol Commun. 2022 Mar 28;10:40. doi: 10.1186/s40478-022-01343-6 (PMC8961908; doi:10.1186/s40478-022-01343-6)
Supplement: Supplementary file 2 — Additional file 2: Supplementary Figs. 1–6. [file 40478_2022_1343_MOESM2_ESM.pdf]

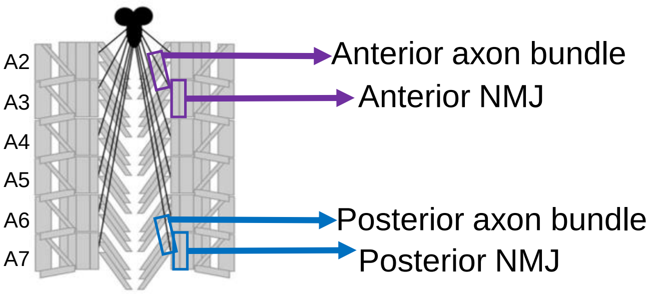

Supplementary fig. 1. Synaptic bouton number and composition is unaffected by loss of Arl6IP1. In *Drosophila* larva, the central nervous system is positioned anteriorly and motor axons exit the ventral nerve cord to innervate the body wall muscles. The schematic diagram illustrates neuronal innervation of segments A2 - A7 in L3 stage *Drosophila* larvae. In this study, unless otherwise stated, we imaged and analysed the longest larval motor neurons i.e. synaptic boutons from NMJs synapsing in segment A7 (posterior NMJ) and motor axons passing through segment A7 (posterior axons).

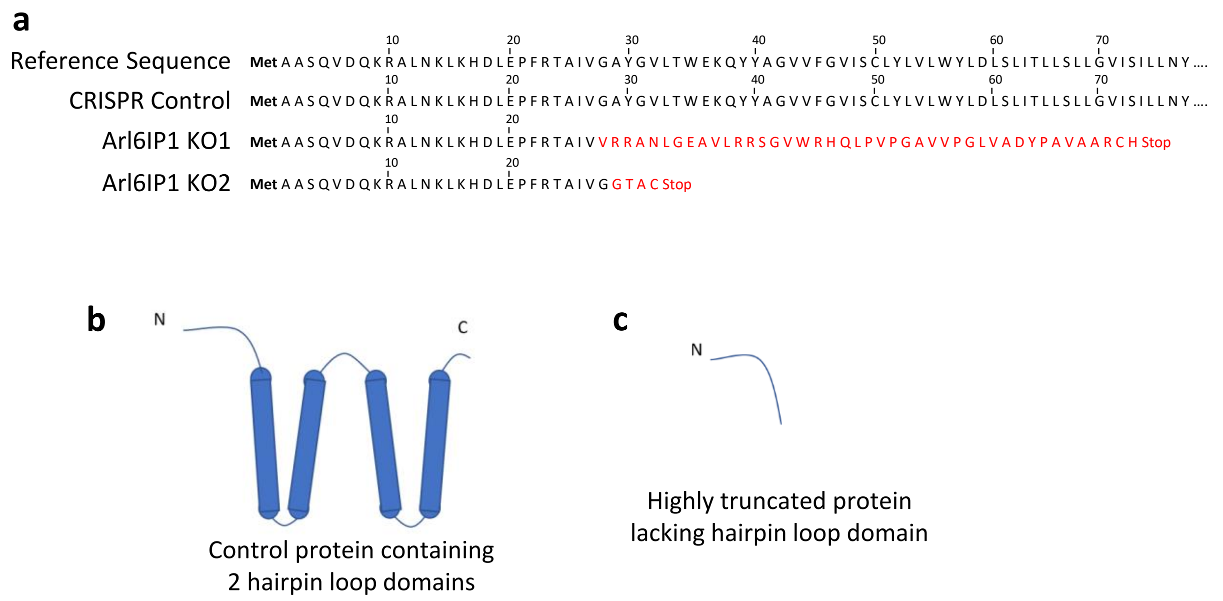

Supplementary fig. 2. CRISPR/Cas9 gene editing of *Drosophila* Arl6IP1 gene. (**a**). The predicted protein sequence resulting from sequence validated Arl6IP1 mutations. WT sequence is indicated in black. Indels induced by CRISPR/Cas9 editing induce frameshift mutations (indicated in red) from amino acid 27 and 26 in KO1 and KO2 respectively. Diagrammatic representation of the Arl6IP1 protein produced in CRISPR Control (**b**) and both Arl6IP1 KO lines (**c**).

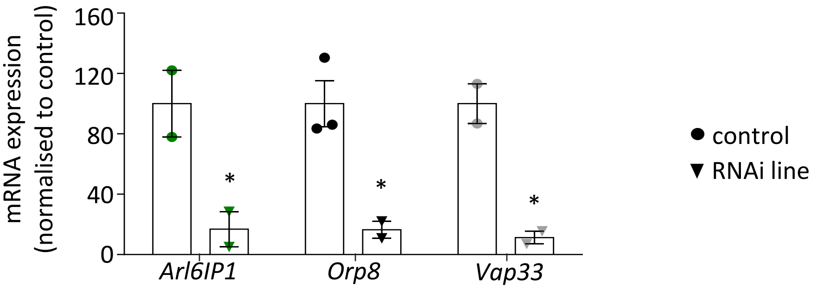

Supplementary fig. 3. Validation of Arl6IP1, Orp8 and Vap33 knockdown *in vivo*. Graph represents mean ± SEM real-time PCR analysis of Arl6IP1, Orp8 and Vap33 mRNA expression in control and RNAi *Drosophila*. $n = 2 - 3$ samples. Statistical analysis consist of two-way ANOVA with Bonferroni's multiple comparisons test.

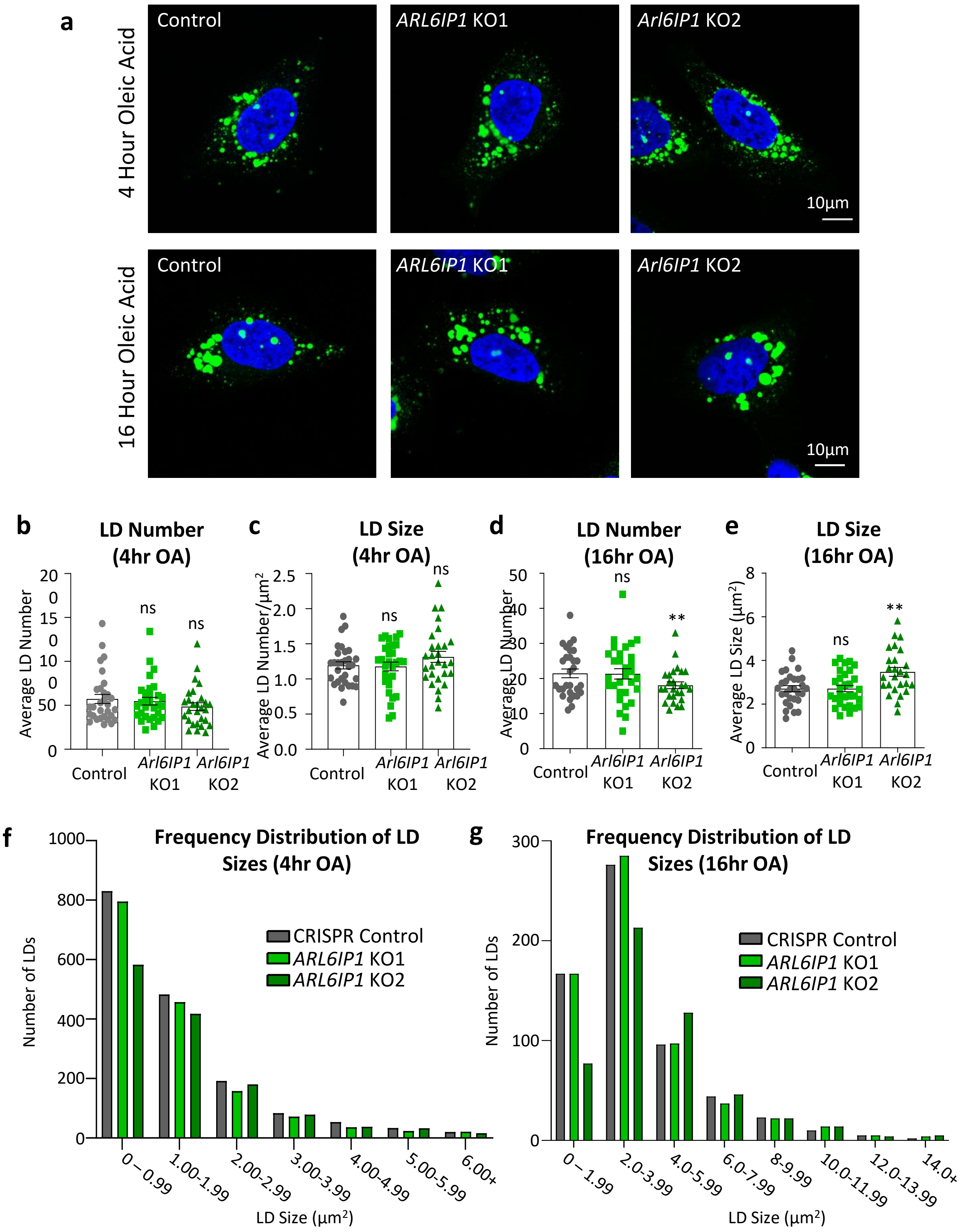

Supplementary fig. 4. Oleic acid (OA) induced lipid droplet formation is not disrupted by loss of ARL6IP1. (**a**) Representative confocal images of LD540 stained LDs (green) in control and *ARL6IP1* knockout (KO) cells treated for 4 or 16 hours with OA. Average LD number (**b** and **d**) or size (**c** and **e**) is not consistently altered in *ARL6IP1* KO cells compared to controls. Graphs represent averages from individual cells. N = 26 - 30 cells per genotype from 3 independent experiments. (**f** and **g**) Frequency distribution of LD sizes showing all LDs measured. Statistics consist of one-way ANOVA with Dunnett's post-hoc tests.

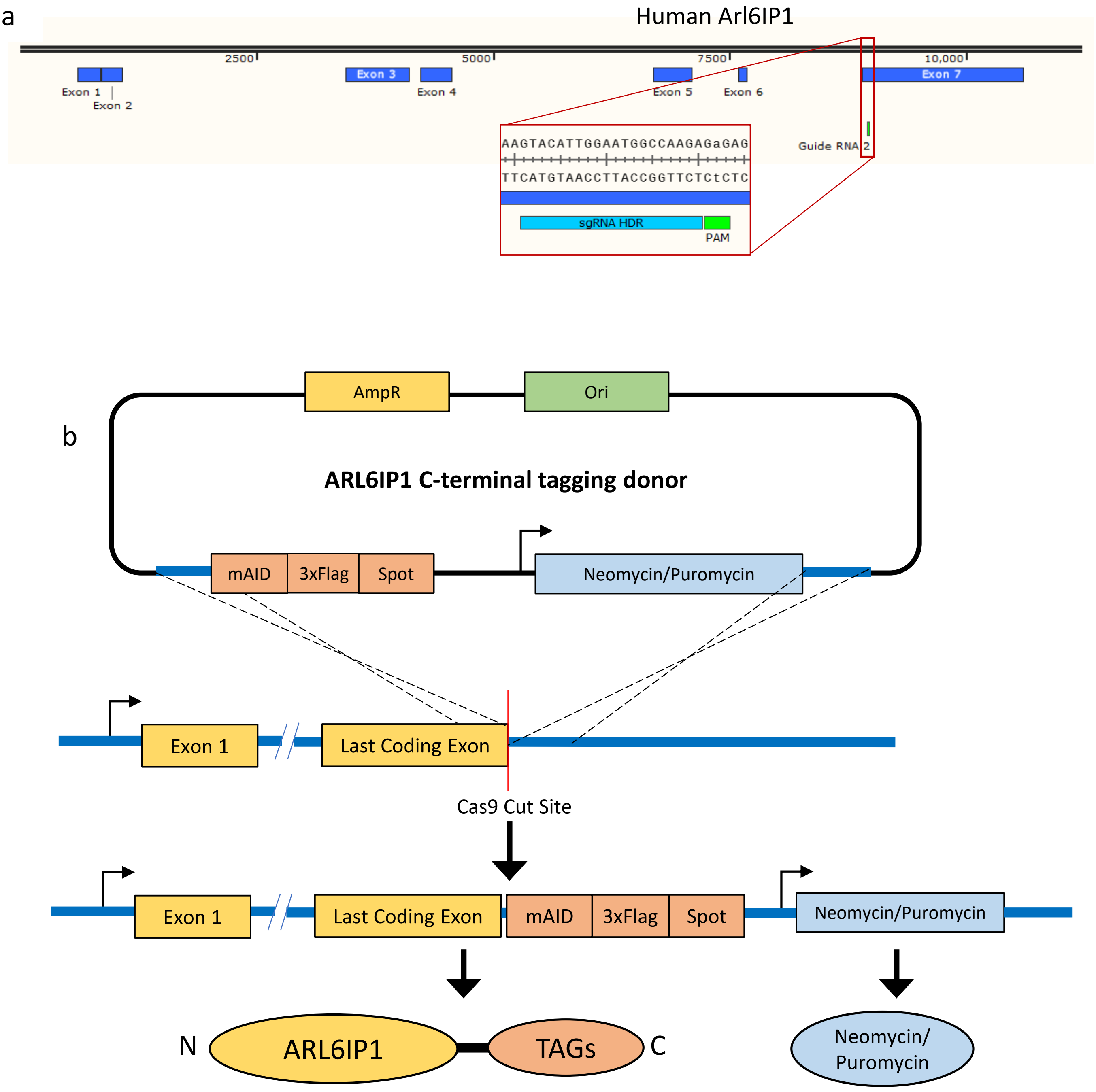

Supplementary fig. 5. Schematic diagram of donor vector construction to endogenously tag human ARL6IP1 in U-2 OS cells. (**a**) A sgRNA was designed to the C-terminal end of human ARL6IP1 to introduce a double stranded break. (**b**) A dsDonor DNA was designed to act as a template for homology directed repair and introduce desired epitope tags.

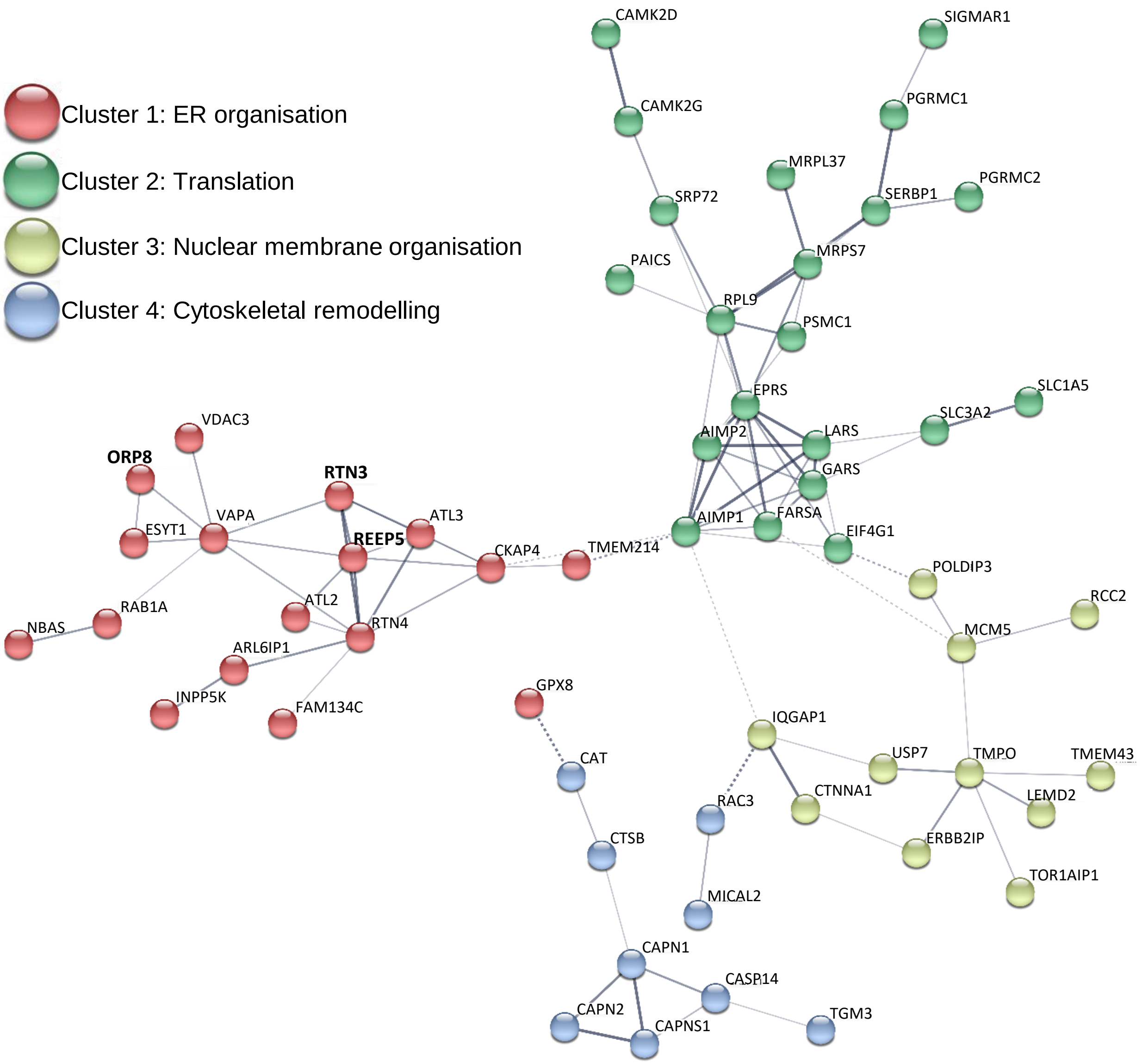

Supplementary fig. 6. Analysis of the ARL6IP1 interactome. ARL6IP1 protein-interaction network was generated and visualised using STRING (https://string-db.org). Network proteins are represented by coloured round nodes. Proteins in bold were validated as ARL6IP1 interactors by independent co-immunoprecipitation and co-localisation experiments in this study. Functional and physical associations between two proteins (edges) are represented by grey lines with line thickness indicating the strength of data support. Associations were accepted if STRING score >0.40. Proteins are coloured according to their grouping in one of four clusters as identified by k means clustering. Legend indicates known or predicted biological process for each cluster.
