# Supplementary material for: Liver X receptor-agonist treatment rescues degeneration in a Drosophila model of hereditary spastic paraplegia
Source: Acta Neuropathol Commun. 2022 Mar 28;10:40. doi: 10.1186/s40478-022-01343-6 (PMC8961908; doi:10.1186/s40478-022-01343-6)
Supplement: Supplementary file 3 — Additional file 3: Supplementary Table 1. List of proteins identified as likely interactors of human ARL6IP1. This list of proteins was generated from the proteins pulled down with ARL6IP1-FLAG. Those included in this list had a spectral intensity abundance ratio (Arl6IP1-FLAG:Control) greater than 1.2 and were not identified in the CRAPome as non-specific binding partners within U-2 OS cells incubated with FLAG M2 affinity beads [48]. GO analysis was conducted to determine the localisation of ARL6IP1 binding partners and shown are proteins identified to have significant enrichment within ER, ER membrane or ER tubular network as determined by D = DAVID, P = Panther, FA = FuncAssociate, E = Enrichr. Proteins in bold were validated as ARL6IP1 interactors by independent co-immunoprecipitation and co-localisation experiments in this study. PSM: peptide spectrum matches. [file 40478_2022_1343_MOESM3_ESM.pdf]

Accession	Gene Symbol	Description	# PSMs	Abundance Ratio: (ARL6IP1) / (control)	ER enrichment
Q15041	ARL6IP1	ADP-ribosylation factor-like protein 6-interacting protein 1	111	100	P, D
Q9NQC3	RTN4	Reticulon-4	19	49.444	P, D, FA, E
Q6DD88	ATL3	Atlastin-3	44	23.86	P, D, FA, E
Q9BT40	INPP5K	Inositol polyphosphate 5-phosphatase K	10	20.053	P, D, FA
O15173	PGRMC2	Membrane-associated progesterone receptor component 2	11	16.951	
**O95197**	**RTN3**	**Reticulon-3**	**6**	**15.667**	**P, D, FA**
Q13557	CAMK2D	Calcium/calmodulin-dependent protein kinase type II subunit delta	15	15.575	P, D, FA
Q96N66	MBOAT7	Lysophospholipid acyltransferase 7	4	14.16	P, D, FA
Q8NHH9	ATL2	Atlastin-2	28	13.616	P, D, FA
Q8TED1	GPX8	Probable glutathione peroxidase 8	5	13.539	P
Q9Y575	ASB3	Ankyrin repeat and SOCS box protein 3	5	12.803	
Q15758	SLC1A5	Neutral amino acid transporter B(0)	4	12.582	
O00264	PGRMC1	Membrane-associated progesterone receptor component 1	11	10.874	P, D, FA
Q9BTV4	TMEM43	Transmembrane protein 43	9	10.381	P
Q07065	CKAP4	Cytoskeleton-associated protein 4	25	10.047	P, D, FA
Q86VR2	FAM134C; RETREG3	Reticulophagy regulator 3	10	9.83	
Q99720	SIGMAR1	Sigma non-opioid intracellular receptor 1	3	9.246	P, D, FA
P22234	PAICS	Multifunctional protein ADE2	22	8.552	
Q15043	SLC39A14	Zinc transporter ZIP14	11	8.543	D
Q93009	USP7	Ubiquitin carboxyl-terminal hydrolase 7	12	8.379	
P08195	SLC3A2	4F2 cell-surface antigen heavy chain	13	8.168	
A2RRP1	NBAS	Neuroblastoma-amplified sequence	4	7.191	P, D, FA
P07384	CAPN1	Calpain-1 catalytic subunit	5	6.821	
O94851	MICAL2	[F-actin]-monooxygenase MICAL2	11	6.547	
Q12904	AIMP1	Aminoacyl tRNA synthase complex-interacting multifunctional protein 1	3	6.471	P, D, FA
Q9Y277	VDAC3	Voltage-dependent anion-selective channel protein 3	6	5.523	
P60763	RAC3	Ras-related C3 botulinum toxin substrate 3	2	5.17	
P35080	PFN2	Profilin-2	3	5.107	
Q6NUQ4	TMEM214	Transmembrane protein 214	5	4.867	P, D, FA
P04632	CAPNS1	Calpain small subunit 1	8	4.107	

Q13555	CAMK2G	Calcium/calmodulin-dependent protein kinase type II subunit gamma	16	3.87	P, D, FA
Q8NC56	LEMD2	LEM domain-containing protein 2	4	3.824	P
Q9BSJ8	ESYT1	Extended synaptotagmin-1	56	3.714	P, D, FA
P41250	GARS	Glycine--tRNA ligase	3	3.633	
Q9BTD8	RBM42	RNA-binding protein 42	4	3.522	
Q9Y2R9	MRPS7	28S ribosomal protein S7, mitochondrial	9	3.455	
P33992	MCM5	DNA replication licensing factor MCM5	14	3.453	
**Q00765**	**REEP5**	**Receptor expression-enhancing protein 5**	**6**	**3.323**	**P,FA,E**
Q9Y285	FARSA	Phenylalanine--tRNA ligase alpha subunit	7	3.121	
P21980	TGM2	Protein-glutamine gamma-glutamyltransferase 2	10	2.978	P, D, FA
**Q9BZF1**	**OSBPL8**	**Oxysterol-binding protein-related protein 8**	**2**	**2.806**	**P, D, FA**
Q9P2J5	LARS	Leucine--tRNA ligase, cytoplasmic	4	2.742	P, FA
P07814	EPRS	Bifunctional glutamate/proline--tRNA ligase	35	2.604	
P32969	RPL9	60S ribosomal protein L9	27	2.105	
P04040	CAT	Catalase	14	1.969	P, D, FA
P62191	PSMC1	26S proteasome regulatory subunit 4	16	1.968	
P35221	CTNNA1	Catenin alpha-1	82	1.956	
Q13155	AIMP2	Aminoacyl tRNA synthase complex-interacting multifunctional protein 2	3	1.953	
Q9P2N5	RBM27	RNA-binding protein 27	5	1.874	
Q96RT1	ERBB2IP	Erbin	20	1.854	
Q9P0L0	VAPA	Vesicle-associated membrane protein-associated protein A	7	1.722	P, D, FA
P42167	TMPO	Lamina-associated polypeptide 2, isoforms beta/gamma	25	1.647	
O60825	PFKFB2	6-phosphofructo-2-kinase/fructose-2,6-bisphosphatase 2	20	1.494	
Q04637	EIF4G1	Eukaryotic translation initiation factor 4 gamma 1	18	1.457	
Q9P258	RCC2	Protein RCC2	9	1.427	
Q08188	TGM3	Protein-glutamine gamma-glutamyltransferase E	30	1.416	
P07858	CTSB	Cathepsin B	2	1.403	
O76094	SRP72	Signal recognition particle subunit SRP72	4	1.398	P, D, FA
P46940	IQGAP1	Ras GTPase-activating-like protein IQGAP1	25	1.393	
Q12931	TRAP1	Heat shock protein 75 kDa, mitochondrial	4	1.376	
Q9BZE1	MRPL37	39S ribosomal protein L37, mitochondrial	4	1.37	

P62820	RAB1A	Ras-related protein Rab-1A	5	1.369	P
Q13509	TUBB3	Tubulin beta-3 chain	150	1.346	
Q9BY77	POLDIP3	Polymerase delta-interacting protein 3	28	1.341	
Q8NC51	SERBP1	Plasminogen activator inhibitor 1 RNA-binding protein	6	1.293	
Q5JTV8	TOR1AIP1	Torsin-1A-interacting protein 1	9	1.293	
P17655	CAPN2	Calpain-2 catalytic subunit	6	1.278	D, P, FA
P31944	CASP14	Caspase-14	33	1.243	
P25311	AZGP1	Zinc-alpha-2-glycoprotein	21	1.218	

Supplementary Table 1. List of proteins identified as likely interactors of human ARL6IP1. This list of proteins was generated from the proteins pulled down with ARL6IP1-FLAG. Those included in this list has a spectral intensity abundance ratio (Arl6IP1-FLAG:Control) greater than 1.2 and were not identified in the CRAPome as non-specific binding partners within U-2 OS cells incubated with FLAG M2 affinity beads [48]. GO analysis was conducted to determine the localisation of ARL6IP1 binding partners and shown are proteins identified to have significant enrichment within ER, ER membrane or ER tubular network as determined by D = DAVID, P = Panther, FA = FuncAssociate, E = Enrichr. Proteins in bold were validated as ARL6IP1 interactors by independent co-immunoprecipitation and co-localisation experiments in this study. PSM: peptide spectrum matches.
